# Supplementary material for: Impact of the COVID-19 pandemic on utilisation of facility-based essential maternal and child health services from March to August 2020 compared with pre-pandemic March–August 2019: a mixed-methods study in North Shewa Zone, Ethiopia
Source: BMJ Open. 2022 Jun 3;12(6):e059408. doi: 10.1136/bmjopen-2021-059408 (PMC9170798; doi:10.1136/bmjopen-2021-059408)
Supplement: Author's manuscript [file bmjopen-2021-059408.draft_revisions.pdf]

BMJ Open is committed to open peer review. As part of this commitment we make the peer review history of every article we publish publicly available.

When an article is published we post the peer reviewers' comments and the authors' responses online. We also post the versions of the paper that were used during peer review. These are the versions that the peer review comments apply to.

The versions of the paper that follow are the versions that were submitted during the peer review process. They are not the versions of record or the final published versions. They should not be cited or distributed as the published version of this manuscript.

BMJ Open is an open access journal and the full, final, typeset and author-corrected version of record of the manuscript is available on our site with no access controls, subscription charges or pay-per-view fees (http://bmjopen.bmj.com).

If you have any questions on BMJ Open's open peer review process please email info.bmjopen@bmj.com

BMJ Open

Impact of COVID-19 Pandemic on Utilization of Facility-Based Essential Maternal and Child Health Services in North Shewa Zone, Ethiopia

Journal:	*BMJ Open*
Manuscript ID	bmjopen-2021-059408
Article Type:	Original research
Date Submitted by the Author:	22-Nov-2021

Complete List of Authors:	Bekele, Chalachew; St Paul's Hospital Millennium Medical College, Birhan HDSS<br>Bekele, Delayehu; St Paul's Hospital Millennium Medical College Department of Obstetrics and Gynecology<br>Hunegnaw, Bezawit; St Paul's Hospital Millennium Medical College, Department of Pediatrics and Child Health<br>Van Wickle, Kimiko; Harvard University T H Chan School of Public Health, Epidemiology<br>Gebremeskel, Fanos ; St Paul's Hospital Millennium Medical College, Birhan HDSS<br>Korte, Michelle ; Harvard University T H Chan School of Public Health, Department of Global Health and Population<br>Tedijanto, Christine ; University of San Francisco, Francis I. Proctor Foundation<br>Taddesse, Lisanu; Ethiopian Public Health Institute<br>Chan, Grace; Harvard Medical School, Boston Children's Hopsital, Department of Pediatrics; Harvard University T H Chan School of Public Health, Epidemiology

Keywords:	COVID-19, Paediatric infectious disease & immunisation < PAEDIATRICS, Antenatal < GENETICS, Community child health < PAEDIATRICS, QUALITATIVE RESEARCH

SCHOLARONE™
Manuscripts

I, the Submitting Author has the right to grant and does grant on behalf of all authors of the Work (as defined in the below author licence), an exclusive licence and/or a non-exclusive licence for contributions from authors who are: i) UK Crown employees; ii) where BMJ has agreed a CC-BY licence shall apply, and/or iii) in accordance with the terms applicable for US Federal Government officers or employees acting as part of their official duties; on a worldwide, perpetual, irrevocable, royalty-free basis to BMJ Publishing Group Ltd ("BMJ") its licensees and where the relevant Journal is co-owned by BMJ to the co-owners of the Journal, to publish the Work in this journal and any other BMJ products and to exploit all rights, as set out in our licence.

The Submitting Author accepts and understands that any supply made under these terms is made by BMJ to the Submitting Author unless you are acting as an employee on behalf of your employer or a postgraduate student of an affiliated institution which is paying any applicable article publishing charge ("APC") for Open Access articles. Where the Submitting Author wishes to make the Work available on an Open Access basis (and intends to pay the relevant APC), the terms of reuse of such Open Access shall be governed by a Creative Commons licence – details of these licences and which Creative Commons licence will apply to this Work are set out in our licence referred to above.

Other than as permitted in any relevant BMJ Author's Self Archiving Policies, I confirm this Work has not been accepted for publication elsewhere, is not being considered for publication elsewhere and does not duplicate material already published. I confirm all authors consent to publication of this Work and authorise the granting of this licence.

For peer review only

Impact of COVID-19 Pandemic on Utilization of Facility-Based Essential Maternal and Child Health Services in North Shewa Zone, Ethiopia

Authors

Chalachew Bekele, BSc MPH[1], Delayehu Bekele, MD MPH[2], Bezawit M. Hunegnaw, MD MPH[3], Kimiko Van Wickle, BSC MSPH[4], Fanos Ashenafi, BSc MSc[1], Michelle Korte, MPH[5], Christine Tedijanto, PhD[6], Lisanu Tadesse, MD MPH[7], Grace J. Chan, MD MPH PhD[3,4,8]

Affiliations

[1]Birhan HDSS, St. Paul's Hospital Millennium Medical College, Addis Ababa, Ethiopia
[2]Department of Obstetrics and Gynecology, St. Paul's Hospital Millennium Medical College, Addis Ababa, Ethiopia
[3]Department of Pediatrics and Child Health, St. Paul's Hospital Millennium Medical College, Addis Ababa, Ethiopia
[4]Department of Epidemiology, Harvard T.H. Chan School of Public Health, Boston, MA, USA
[5]Department of Global Health and Population, Harvard T.H. Chan School of Public Health, Boston, MA, USA
[6]Francis I. Proctor Foundation, University of California San Francisco, San Francisco, CA, USA
[7]HaSET MNCH research program, Addis Ababa, Ethiopia.
[8]Division of Medical Critical Care, Boston Children's Hospital, Harvard Medical School Boston, MA, USA

Corresponding Author

Grace J Chan, MD MPH PhD
677 Huntington Ave, Kresge 913, Boston, MA 02115
grace.chan@hsph.harvard.edu

Word Count: 4,555 (Excluding title, Abstract, figure, table and references)

Abstract word count = 296 (Excluding titles)

Abstract

Introduction: Ethiopia registered its first case of COVID-19 on March 13, 2020. We aimed to assess maternal, newborn, and child health care (MNCH) utilization during the first six months of the COVID-19 pandemic, as well as potential barriers and enablers of service utilization from health care providers and clients.

Methods: Mixed study design was conducted as part of the Birhan Health and Demographic Surveillance System in Ethiopia. The trend of service utilization during the first six months of COVID-19 was compared to corresponding time and data points of the preceding year.

Result: Service utilization of new family planning visits (43.2 to 28.5/month, p = 0.014) and sick under five child visits (225.0 to 139.8/month, P = 007) declined during the initial six months of the pandemic compared to the same period in the preceding year. Antenatal and postnatal care visits, facility delivery rates, and child routine immunization visits also decreased although this did not reach statistical significance. Interviews with health care providers and clients highlighted several barriers to service utilization during COVID-19, including fear of disease transmission, economic hardship, and transport service disruptions and restrictions. Enablers of service utilization included communities' decreased fear of COVID-19, and awareness-raising activities.

Conclusion: Provision of essential MNCH services is crucial to ascertain favorable maternal and child health outcomes. In low- and middle-income country settings like Ethiopia, health systems might be fragile to withstand the caseloads and priority setting due to the pandemic. Our study presents early findings on the utilization of MNCH services that were maintained except sick child and new family planning visits. Government leaders, policy makers, and clinicians who wish to improve the resilience of their health system will need to continuously monitor service utilization and clients' evolving concerns during the pandemic to prevent increases in maternal and child morbidity and mortality.

What is already known?

Facility-based essential MNCH services utilization decreased during the initial phase of the pandemic and similarly facility-based healthcare utilizations were reduced in the 2014-2015 Ebola outbreak in west Africa.

What are the new findings?

Facility based essential MNCH services such as antenatal care, postnatal care, family planning, facility deliveries, routine immunization and repeat family planning utilization were maintained in the initial six month of the pandemic unlike other similar studies elsewhere.

What do the new findings imply?

In light of a pandemic, essential MNCH services such as antenatal and postnatal care, family planning, facility deliveries, repeat family planning and routine immunizations can be sustained in a health system. More attention may be given to better understand the reduction of sick under five visits. Further research can be conducted on the utilization of essential MNCH services on maternal and child health outcomes. Our results emphasize the importance of health systems and clinicians to sustain the resilience of their health system. Among those the Ministry of Health

(MoH) directive to avail MNCH services in all facilities during the pandemic and the maturity level of some programs (Even though new family planning utilizers are limited, they know the benefit and would want to continue the repeat family planning utilization, benefits of facility delivery, routine immunization, antenatal care and postnatal care).

Strengths and limitations of this study

Strengths of the study:

- ✓ We present primary data on service utilization during the early months of the pandemic in an area of Ethiopia, one of the agrarian regions, which is generalizable to 80% of the country's population.
- ✓ The mixed methods approach integrated both quantitative service utilization coverage data with sociocultural, contextual, and exploratory qualitative to better understand our findings and reasons for changes in service utilization.
- ✓ The study highlights success stories in community-based care and government leadership for key services like routine immunization that may benefit other settings.

Limitation of the study:

- ✓ Our study focused on service utilization and may not have been powered to detect significant differences. Furthermore, we focused on coverage of service utilization as the primary outcome rather than mortality or morbidity.
- ✓ We do not have detailed data on service provision (e.g., which services were restricted and for how long, in what manner).
- ✓ There is the potential of recall bias were possible limitation since qualitative data was collected three months later than the initial six months of the pandemic (March to August 2020).

INTRODUCTION

The World Health Organization (WHO) declared coronavirus disease-2019 (COVID-19) a global pandemic on March 11, 2020[1] and the first case of COVID-19 in Ethiopia was registered on March 13, 2020.[2] Multiple preventive measures focusing on social distancing and wearing masks were undertaken.[3] Some health facilities were assigned as COVID-19 isolation and quarantine centers, and many suspended conducting elective surgeries and select outpatient services. This increasing burden of managing COVID-19 on health facilities and health care providers leaves the health system overstretched, challenging its ability to operate effectively. As shown during the 2014-2015 Ebola outbreak in west Africa, when health systems are overwhelmed by outbreaks, mortality from vaccine-preventable and other treatable diseases can increase dramatically.[4,5]

Well-organized and prepared health systems can continue to provide equitable access to essential services throughout an emergency[6], but health systems in developing countries are often fragile when affected by emergencies such as pandemics. Accordingly, the WHO advises that countries should identify and prioritize essential services like routine vaccination, reproductive health services including care during pregnancy and childbirth, and care of young infants and

For peer review only - http://bmjopen.bmj.com/site/about/guidelines.xhtml

older adults in their efforts to maintain continuity of service delivery and make strategic shifts to ensure that increasingly limited resources provide maximum benefit for the population.[7] The disruption of services and diversion of resources away from essential sexual and reproductive health care due to the prioritization of the COVID-19 response are expected to increase risks of maternal and child morbidity and mortality.[8]

The Ministry of Health (MOH) of Ethiopia focused on prevention and control of the pandemic. Possible shifts of the health workforce and the health system towards the COVID-19 response may contribute towards low utilization of routine services. For example, "half sit" policies (29 March 2020) decreased maximum occupancy on public transit, thus cutting the number of seats in half and increasing the cost of transportation when traveling within the region while transportation between regions was paused completely. Additionally, school closures (March 2020), declaration of state of emergency (08 April 2020), awareness campaigns about the pandemic, and case reports, both suspected and confirmed cases, may have all contributed to growing fear of exposure to COVID-19, especially for patients visiting health facilities.

A modelling study of essential maternal and child health interventions across 118 low- and middle-income countries over a 6-month period estimated reduction of services by 9.8–18.5% and 39.3–51.9% in the least and most severe scenarios, respectively,[9] due to the pandemic. Service reductions have already borne out in several contexts. In China, health service utilization declined significantly after the outbreak and all indicators rebounded beginning in March, but most had not recovered to their pre-COVID-19 levels by June 2020.[10] In Bangladesh, Nigeria, and South Africa, between March and May 2020, the utilization of basic essential MNCH services such as antenatal care (ANC), family planning (FP), and immunization reduced due to lockdowns that triggered fear of contracting COVID-19, shifts of health system focused on managing the pandemic, and resource constraints.[11] During the early phase of the COVID-19 outbreak (March-April 2020) in Rwanda, utilization of ANC, deliveries, postnatal care (PNC) and immunizations significantly declined.[12] Similarly, a study in western Ethiopia showed a significant reduction in mean utilization of ANC, health facility birth, FP, and newborn immunization services between March–June 2019 and March–June 2020.[13]

In Nepal, a qualitative study found that maternity services, immunizations, and supply of essential medicine were the most affected health services during the lockdown. Interruptions were mainly due to the closure of health services at local health care facilities, limited affordability, involvement of private health sectors during the pandemic, fears of COVID-19 transmission among health care workers and within health centers, and disruption of transportation services. Participants expressed frustrations on poor testing, isolation and quarantine services related to COVID-19, and poor accountability from the government at all levels towards health services continuation and management during the COVID-19 pandemic.[14]

To understand these effects COVID-19 pandemic in Ethiopia, the HaSET Maternal and Child Health Research Program assessed trends in MNCH care utilization from March 2019 to August 2020 as well as health care providers' and clients' perceptions on the barriers to and enablers of service provision and utilization during the COVID-19 emergency. This study has paramount importance in filling the evidence gap on MNCH service utilization during COVID-19, both in the

For peer review only - http://bmjopen.bmj.com/site/about/guidelines.xhtml

Ethiopian context and other low- and middle-income countries, to prevent significant damage to the gains achieved in such areas over the past several decades.

METHODS

We conducted the study in eight health facilities, five health centers, and three hospitals (two public and one private), as part of the Birhan Health and Demographic Surveillance System (HDSS) in North Shewa Zone, Amhara Region, Ethiopia. Those facilities provide essential MNCH services for both rural (majority) and urban populations coming from HDSS catchment and non-catchment areas.

The health centers provide ANC, PNC, delivery, abortion, routine immunization (RI), integrated management of neonatal and childhood illness (IMNCI), and FP. Each health center also has a minimum of five service extension health posts mainly for FP and RI in each kebele (the lowest simplest administration unit), and each health post sends activity reports to health centers monthly. Two public hospitals (one primary and one referral) and the remaining one private general hospital also provide the above-mentioned essential MNCH services, except for RI, which is given mainly in health centers and catchment health posts.

Mixed qualitative and quantitative methods were employed. For the quantitative part of the study, a facility-based cross-sectional survey was conducted with MNCH healthcare providers to assess the impact of COVID-19 on essential MNCH service provision or utilization and provider-side barriers to service provision and utilization in Birhan catchment health facilities. Healthcare utilization time-series data from each facility was retrospectively collected and analyzed to understand the impact of COVID-19. In addition to this, a phenomenological qualitative design utilizing in-depth interviews was implemented to assess client and provider side barriers and enablers to service provision/utilization in Birhan catchment health facilities.

Birhan HDSS catchment health facilities' medical records and monthly facility reports, interviews with health care providers working in the MNCH department, and interviews with women who delivered at home and facility, had ANC follow up, and who missed follow up were the data sources. All Birhan catchment health facilities were sampled for service statistics and health care providers who were working in essential MNCH departments and available at the time of visit were asked for respective sections. For the qualitative data, purposive sampling was implemented, and in-depth interviews were conducted until theoretical saturation was reached.

To assess health care providers' perceptions on possible barriers to service utilization during the time of COVID-19, data were collected from interviews with health providers working in respective MNCH departments and facility and department heads. Retrospective facilities service statistics were collected over an 18-month period from March 2019 to August 2020 using Computer Assisted Field Editing (CAFE). Data was abstracted by uniformly structured questionnaires and entered to Open Data kit (ODK) collect and uploaded to ODK aggregate. The facilities' monthly reports and medical registers data were collected separately. The monthly reports include services given in the health posts that are extension sites for the health center, but the facility registers are exclusively for services given in the health centers.

An interview guide with open-ended questions was used to elicit the qualitative information from informants. Face-to-face interviews were conducted in the facilities with women who visited facilities during COVID-19 and women who delivered at home. Women who missed an ANC follow up were interviewed by phone. With the permission of the respondent, all interviews were recorded, and all recorded data was transcribed for further analysis. To ensure the safety of the data collectors and participants, masks were worn, and social distancing practices were implemented during training and data collection from 2 – 20 November 2020.

The extracted data was exported to Stata 17.0 for analysis and the average MNCH services uptake was calculated each month to quantify the changes pre – COVID-19 (March to August 2019) and during the COVID-19 (March to August 2020) pandemic. For the purposes of analysis, March to August 2019 and March to August 2020 were considered as pre-COVID-19 and COVID-19 periods, respectively. To avoid the effect of missing and partially filled values, analogous months data from the same facility were excluded from the data analysis. Finally, an independent sample t-test was done to compare pre-COVID-19 and COVID-19 time months. This analysis was repeated for the initial two-months (March to April 2020) of the pandemic and the analogous period, March to April 2019, to examine changes in service utilization at the onset of the COVID-19 pandemic and a significance level of $\alpha=0.05$ was used for all statistical tests.

In addition to the quantitative metrics listed above, English language transcript data was entered in Dedoose software for qualitative data analysis. After familiarization with the data, the content of the data was coded line by line for thematic analysis following a framework theory approach to describe and interpret health providers' and communities' perceptions on barriers and enablers to MNCH service provision. The framework approach involves using some pre-assigned themes to initially categorize data while also adjusting and iterating the coding scheme to accommodate newly emergent themes, sub-themes, and categories through inductive interpretation.[15] Coded data was examined for potential relationships and themes were also assessed across relevant participant demographic categories to understand different user perspectives. Findings were described under pre-assigned and newly emerged themes.

Ethic Statement

This study involves human participants and was approved by Ethics Review Board (IRB) of Saint Paul's Hospital Millennium Medical college (SPHMMC) and Harvard T.H. Chan School of Public Health (HSPH) (IRB20-1574). To extract MNCH service statistics, permission was obtained from individual health facilities and individual verbal consent was obtained from respondents.

Patient and Public Involvement

Meeting was restricted during protocol development and study period due to COVID-19 pandemic and it was not possible to involve clients or the public in the design, study, reporting and dissemination plans of our research.

For peer review only - http://bmjopen.bmj.com/site/about/guidelines.xhtml

RESULTS

For the quantitative section of the study, data were abstracted from a total of eight health facilities (three hospitals and five health centers) and interviews with 103 healthcare providers working in the MNCH units of the facilities. In addition to these, ten facility or MNCH department heads and nine women (pregnant and delivered in the time of COVID-19) were asked open-ended questions.

Maternal health facility visits for ANC, PNC, facility delivery, and abortion-related services decreased in the time of COVID; however, we do not see a statistically significant change. The FP services utilization in the health centers and hospitals declined from 105.5 visits per month to 66.5 visits per month ($p < 0.05$) after the onset of the pandemic and within the subset of FP visits, repeat and unclassified FP visits significantly declined while new FP visits did not change. When combining health facilities with community health post data, the new FP services declined significantly from 43.2 visits per month to 28.5 visits per month ($p = 0.029$) but no significant changes in repeat, unclassified, and mean FP visits.

Declines in service utilization were found among sick child visits, which was defined as a facility visit for sick children under five years old. The mean number of IMNCI visits for sick children under 5 years old declined from 225.0 visits per month in 2019 to 139.8 visits per month in 2020 ($p = 0.014$). This significant relationship persists for two age stratifications of IMNCI visits (2 months to under 2 years, and 2 years to under 5 years). On the other hand, there was no significant change in child visits for routine immunizations, including BCG, OPV-0, pentavalent (DPT-HepB-HIP) and measles vaccinations.

Table 1. *Comparing essential MNCH service utilization over six months between COVID-19 (Mar-Aug 2020) and analogous pre-COVID-19 (Mar-Aug 2019) periods.*

Visit Type	Mean number of visits/month over six months		t-statistic	p-value	Lower p-value†	Upper p-value††	Paired observations
	2019	2020					
**I. Maternal visit**	**376.3**	**321.2**	**1.11**	**0.270**	**0.865**	**0.135**	**48**
1. Antenatal care	208.9	181.7	0.79	0.433	0.784	0.216	40
2. Postnatal care	26.6	19.8	1.44	0.155	0.922	0.078 *	30
3. Facility delivery	90.7	84.2	0.29	0.776	0.612	0.388	41
4. Abortion-related services	11.8	9.8	0.56	0.578	0.711	0.289	34
5. Combined FP services in HP, HC and hospitals	313.3	273.4	0.82	0.415	0.792	0.207	47
5.1 New FP services	43.2	28.5	1.22	0.029	0.986	0.014 **	47
5.2 Repeat FP services	270.2	244.9	0.57	0.567	0.716	0.284	47
6. FP services in HCS and hospital	105.5	66.5	1.99	0.051	0.974	0.026 **	33
6.1 New FP services	8.9	7.1	0.84	0.406	0.797	0.203	33

6.2 Repeat FP services	96.5	59.3	1.03	0.046	0.977	0.023 **	33
6.3 Unclassified FP services	17.7	1.3	1.12	0.039	0.981	0.019 **	26
**II. Sick child visit (0-5years))**	**225.0**	**139.8**	**1.51**	**0.014**	**0.993**	**0.007 *****	**46**
1. MNCI Visit (< 2 months)	10.8	7.7	0.82	0.412	0.794	0.206	46
2. IMNCI Visit (2 months – 2 year)	101.6	50.4	1.68	0.009	0.996	0.004 ***	46
3. IMNCI Visit (2 year – 5 year)	111.6	81.8	1.15	0.034	0.983	0.017 **	46
**III. Routine Immunization visit**	**37.0**	**36.8**	**0.02**	**0.982**	**0.509**	**0.491**	**23**
1. BCG Vaccine	31.4	36.5	-0.39	0.701	0.350	0.650	30
2. Oral Polio (0) Vaccine	3.2	1.0	0.88	0.384	0.808	0.192	23
3. Pentavalent (DPT-HepB-HIP) (all types)	100.4	101.5	-0.05	0.958	0.479	0.521	30
4. Measles – 1	10.3	27.5	-1.99	0.051	0.026	0.974	30
5. Vitamin A Dose	8.6	5.7	0.67	0.506	0.747	0.253	14
**Other types of visits**							
All visits	2568.9	2606.7	-0.05	0.956	0.478	0.522	48
Adult Outpatient Visit	2121.2	2239.7	-0.17	0.868	0.434	0.566	44

* $p < 0.10$ ** $p < 0.05$ *** $p < 0.01$, † Lower-tailed test: mean number of visits H_1: $\mu_{2019} < \mu_{2020}$, †† Upper-tailed test: mean number of visits H_1: $\mu_{2019} > \mu_{2020}$

"Ninety-one healthcare providers who were working in maternal, newborn and child health were asked about the client flow during COVID-19. Sixty-seven percent of the health care providers (HCPs) perceived that client flow decreased and 31% of them considered the same. Qualitative interviews also supported the observed decrease in client flow, with descriptions of sharper contractions in service utilization in the first couple of months after the onset of the pandemic but resumed to approximately normal levels over subsequent months. To explore the perception of lower service utilization during the initial couple of months of COVID-19 by clients of the health system and HCPs, data on the initial two pre-COVID (March to April 2019) months were compared with analogous COVID time months (March to April 2020) and there were no statistically significant changes in the number of visits for maternal and childhood visits overall, except sick child visit **(Supp Table 1)**.

Barriers to service provision and utilization during COVID-19

Even though the essential MNCH service utilization was maintained; clients' fear of acquiring the disease from the facility, travel restrictions, increased transportation cost due to the half seat order by the government, and fear of acquiring the disease on the way to the health facility were the main barriers for service utilization perceived by healthcare providers.

Table 2. *Possible barriers to service utilization in the time of COVID-19 based on healthcare providers' perception.*

For peer review only - http://bmjopen.bmj.com/site/about/guidelines.xhtml

Possible barriers to service utilization in the time of COVID-19	Count	%
1. Fear of acquiring the diseases from the facility	97	94
2. Travel restrictions	90	87
3. Increased transportation cost (due to half sit order by the government)	89	86
4. Fear of acquiring the disease on the way to the health facility	86	83
5. Lack of transport to the HP/HC site	72	70
6. Lack of PPE for clients	67	65
7. Clients' perception of limited implementation of protective measures by<br>8. healthcare providers	58	56
9. Healthcare providers advice to stay at home	54	52
10. Limited-service hours or absence of health care workers	17	17
11. Unavailability of ambulance	7	7
12. Unavailability of healthcare providers in facilities to provide outreach services.	7	7
Total eligible respondents	**103**	

These secondhand perspectives from healthcare providers about the barriers that clients face was largely supported by qualitative interviews with clients. Fear of contracting the disease and lack of access to transportation are the most described barriers **(Supp table 2).** Particularly during the first few months after the onset of COVID-19 in Ethiopia and the imposition of travel restrictions and other public health measures like state of emergency, communities fear of acquiring the disease and high levels of public panic were barriers for facility-based services utilization. This fear extended to visiting facilities during the pandemic; community members were afraid of contracting the disease in crowded spaces, public transportation routes to facilities, or at facilities themselves from health care workers or other patients, particularly when they heard of COVID-19 cases present at facilities and this fear often resulted in delayed care-seeking. A woman said that *"I have postponed my follow up at that time for fear of acquiring the disease from health professionals and health centers. The same is true for other clients in our area, and some mothers have received their visit in private clinics as we perceived almost all staff were infected"*.

The economic hardship during COVID-19 prevented some clients from being able to pay for transportation due to transportation restriction with half seats and doubled transportation fee, and other direct or indirect costs of attending facilities. Clients described lacking money to purchase PPE and one HCP noted that the closed market movement affected people's incomes, as reflected by patients delaying treatment until conditions are more severe or defaulting on treatment.

For peer review only - http://bmjopen.bmj.com/site/about/guidelines.xhtml

Lastly, bottlenecks on the health system side provided another barrier to service utilization. Facilities restricted some services at the beginning of the pandemic's onset and clients were unable to access certain services or assumed that services were restricted even after they had resumed, as one HCP suggested. Additionally, multiple clients described fearing that they might be forcibly quarantined or presumed COVID-19-positive if they were to visit facilities and this fear deterred facility visits. HCPs described many challenges related to the under preparedness of the health system for managing suspected cases of COVID-19. Often these challenges manifested through physical infrastructure constraints and a shortage of guidelines for managing quarantine and isolation centers for suspected COVID-19 cases.

Enablers of service provision and utilization during COVID-19

In terms of knowledge of COVID-19, all women had heard about the disease, but a few were in doubt about COVID-19 existence in the area which may be an enabling factor for facility-based service utilization. A client respondent said, "*I do not believe it exists, especially in our area. It might be real / exist in other areas/countries. They just suspect and take everyone into an isolation/quarantine center, but they are healthy and free of any signs and symptoms…*". Some described that COVID-19 could not affect them because God and/or Mary will protect them, citing the importance of prayer as a protective measure.

Facility adaptations like training for healthcare providers, hand washing facilities, physical distancing, and awareness creation and health education which was given by local authorities increased the client awareness on COVID-19 prevention and facility-based service utilization through time **(Supp table 3).**

Understanding Service Utilization Trends during COVID-19

The barriers and enablers highlighted in the interviews interact with each other dynamically, as depicted below. Certain barriers were more substantial than others, particularly fear of the disease, transportation access, and economic-related barriers, while certain pull factors encouraged facility visits, particularly over time as fear subsided, community awareness measures were undertaken, and facilities implemented adaptations to manage both COVID-19 and routine services **(Diagram 01).**

DISCUSSION

We examined the impact of the COVID-19 pandemic on essential MNCH service utilization by analyzing data from health facility records and healthcare providers and patients' perspectives.
In the context of already poor health outcomes, significant reductions in service utilization for maternal and child health may have substantial adverse impacts**.** A modelling study of 118 low- and middle-income countries estimated an additional 12,200-56,700 maternal and 253,500-1,157,000 child deaths using several hypothetical scenarios in which the coverage of essential maternal and child health interventions were reduced by 9.8-51.9% due to the pandemic over 6 months.[9]

For maternal health, FP, ANC, PNC, facility delivery, and abortion services utilization decreased, but the change was not significant during the initial six months of the pandemic. Globally, contraception services were shut down or not accessible,[16] which was also observed in our study resulting in reduced family planning service utilization and a drastic drop in new family planning services. Service utilization for family planning was stable at health posts (community-based clinics that are an extension of health centers) suggesting that utilization of family planning services was more likely to occur when clinics are nearby without extensive travel.

For child health, the number of Integrated Management of Neonatal and Childhood Illness (IMNCI) visits, also referred to as sick child visits, significantly declined by 38%. It is possible that the decrease in child sick visits was related to COVID-19 prevention and control activities. The leading causes of under five years old children morbidity in Ethiopia,[17] acute respiratory illness, fever and diarrhea may have decreased due to school closures (older siblings less exposed), limited interactions with peers in the community, spending more time indoors, mask-wearing at community gatherings, hand washing, physical distancing, and other personal protective equipment and practices. We found that RI remained stable during the initial six months of the pandemic, which was different than the findings from studies in Colombia, India, and Brazil where RI declined during the pandemic.[18-20] The Ethiopian MOH prioritized RI, especially measles, during COVID-19. Ethiopia deploys health extension workers stationed at health posts for community-based services hygiene and sanitation, FP and RI which may have sustained accessibility to these services during COVID-19. It is worth nothing that the source of data for FP and RI in this study included aggregated data from the health management information system (HMIS) at health centers which may be less reliable than directly collecting data from hospital and health centers' records other services like IMNCI and sick child visits. [21] In Ethiopia, during COVID, there was a marked reduction in supply chain distribution of vaccines implying that the RI coverage like decreased nationally.[22]

Overall, with the exception of RI, our findings are similar to other global studies that have also found declines in service utilization during the COVID-19 pandemic. A systematic review of eighty-one studies in twenty countries reported a reduction in health care utilization with a median 37% reduction in overall services, including 42% reduction for visits, 28% for admissions, 31% for diagnostics, and 30% for therapeutics between pandemic and pre-pandemic periods in the initial two months of the pandemic.[23] There was also a reduction in utilization of basic essential MNCH services such as ANC, FP and RI in Bangladesh, Nigeria, Rwanda and South Africa between March and May 2020.[11,12] In a semi-pastoralist area in western Ethiopia, there was a significant reduction in mean utilization of ANC, health facility births, FP, and RI.[13]

The reduction in service utilization was observed in the setting of the communities' experiences of and perceptions toward COVID-19, including misinformation, misconceptions, and doubt. As the pandemic has progressed, service utilization may further decline as fear settles in. At the time of data collection, early in the pandemic, respondents largely described not feeling many tangible impacts of COVID-19 on their daily lives or consequences of lack of adherence to preventive measures, so they went about life as usual. This easing fear of COVID-19 may have enabled women to feel that they could safely attend services, but it also has important implications as the

For peer review only - http://bmjopen.bmj.com/site/about/guidelines.xhtml

pandemic continues, particularly as cases in Ethiopia have risen substantially. Awareness and education campaigns are needed to produce actual behavior change. Moreover, communities' belief that God may protect them from infection indicates the important role of engaging religious leaders as champions in behavior change campaigns. An additional key recommendation is systematically addressing misinformation and doubt to increase population compliance with preventive measures, particularly as Ethiopia faces a rising caseload, increasing prevalence of variants, and a stalled vaccine rollout that may take months or years to reach substantial population coverage. Less than 2% of the population has received at least one dose of COVID-19 vaccine.[24]

Barriers to maternal facility visits included women not wanting to bother anyone, lack of support from healthcare workers, influence of the media,[25] lockdown, fear of contracting the disease,[26] shift of focus towards pandemic, and resource constraints.[11] In addition, women experienced fears of contracting the disease, economic hardship, and lack of access to transportation. Particularly during the first few months after the onset of COVID-19 in Ethiopia, there was an imposition of travel restrictions and other public health measures like state of emergency, and high levels of public panic. In addition, facilities restricted some services at the beginning of the pandemic and clients were unable to access certain services. Multiple clients described fearing that they might be forcibly quarantined or presumed COVID-19-positive if they were to visit facilities; this fear deterred facility visits. While we found that sick child visits and new family planning services were most affected by the pandemic, the declines among other essential services were not as significant indicating hope in service resilience and the ability to introduce rapid and substantial facility adaptations to maintain the health system (e.g., personal protective equipment, infection prevention measures, improved sanitation/hygiene).

Strengths of the study: We present primary data on service utilization during the early months of the pandemic in an area of Ethiopia, one of the agrarian regions, which is generalizable to 80% of the country's population. We leveraged an existing research network, HaSET MNCH research program (www.hasetmch.org), and established field site[27] to rapidly collect data from all available sources. The mixed methods approach integrated both quantitative service utilization coverage data with sociocultural, contextual, and exploratory qualitative to better understand our findings and reasons for changes in service utilization. The study highlights success stories in community-based care and government leadership for key services like routine immunization that may benefit other settings.

Limitation of the study: Our study focused on service utilization and may not have been powered to detect significant differences. Furthermore, we focused on coverage of service utilization as the primary outcome rather than mortality or morbidity. We do not have detailed data on service provision (e.g., which services were restricted and for how long, in what manner). There is the potential of recall bias were possible limitation since qualitative data was collected three months later than the initial six months of the pandemic (March to August 2020).

CONCLUSION

Utilization of essential MNCH services is crucial to achieve favorable health outcomes. In the setting of developing countries like Ethiopia, health systems are often too fragile to withstand the direct increase in volume of patients and indirect health consequences of a pandemic. Our study presents early findings on the decline in the utilization of MNCH services especially in new family planning services and sick child visits. Further study is needed to assess the effect of service utilization decline on MNCH morbidity and mortality. To prevent worsening maternal and child morbidity and mortality as a result of the pandemic, resources are required by government leaders, policy makers, and clinicians to improve the resilience of their health system to continuously monitor service utilization, while at the same time engaging with providers and clients to understand and address their evolving concerns about MNCH service uptake.

ABBREVIATIONS

CAFÉ: Computer Assisted Field Editing, COVID-19: Coronavirus Disease - 2019, DPT: Diphtheria Pertussis and Tetanus, EOC: Emergency Operation center, EPHI: Ethiopian Public Health Institute, FP: Family Planning, HC: Health Center, HCP: Healthcare provider, HDSS: Health and Demographic Surveillance System, HepB: Hepatitis B, HIP: Haemophilus influenzae, HIV/AIDS: Human Immunodeficiency Virus/ Acquired Immune Deficiency Syndrome, HP: Health post, HSPH: Harvard School of Public Health, IRB: Institutional Review Board, MCH: Maternal and Child Health, MNCH: Maternal, Newborn and Child Health, MOH: Ministry of Health, ODK: Open Data Kit, PNC: Postnatal care, RI: Routine immunization, RMNCH: Reproductive, Maternal, Newborn and Children Health, SPHMMC: Saint Paul Hospital Millennium Medical College, W: Women, WHO: World Health organization.

ACKNOWLEDGEMENTS

We acknowledge the kind help and encouragement we received from the zone, woreda and study facilities. Our deepest gratitude goes to the women and health care providers who participated in this study and to all data collectors and supervisors for their dedicated work.

FUNDING

This work is supported by the Bill & Melinda Gates Foundation.

GRANT/AWARD NUMBER

The research is co-funded through INV-006752 HaSET and INV-003612 ANC/PNC Innovations Platform HaSET Ethiopia Partnership.

AVAILABILITY OF DATA AND MATERIALS

The datasets used and/or analyzed during the current study are available from the corresponding author on reasonable request.

COMPETING INTERESTS

The authors declare that they have no competing interests.

For peer review only - http://bmjopen.bmj.com/site/about/guidelines.xhtml

References

1. World, Organization H. *Coronavirus disease (COVID-19) situation report – 162*. 2020.
2. https://www.worldometers.info/coronavirus/
3. Agency EP. *Ethiopia officially declares State of Emergency*. April 9, 2020. https://www.press.et/english/?p=20750#
4. Elston JW, Cartwright C, Ndumbi P, Wright J. The health impact of the 2014-15 Ebola outbreak. *Public Health*. Feb 2017;143:60-70. doi:10.1016/j.puhe.2016.10.020
5. Parpia AS, Ndeffo-Mbah ML, Wenzel NS, Galvani AP. Effects of Response to 2014-2015 Ebola Outbreak on Deaths from Malaria, HIV/AIDS, and Tuberculosis, West Africa. *Emerg Infect Dis*. Mar 2016;22(3):433-41. doi:10.3201/eid2203.150977
6. Organization WH. 2019 novel coronavirus (2019‑nCoV): strategic preparedness and response plan. 20 February 2020.
7. Organization WH. WHO releases guidelines to help countries maintain essential health services during the COVID-19 pandemic. March 30, 2020.
8. Hall KS, Samari G, Garbers S, et al. Centring sexual and reproductive health and justice in the global COVID-19 response. *Lancet*. Apr 11 2020;395(10231):1175-1177. doi:10.1016/s0140-6736(20)30801-1
9. Roberton T, Carter ED, Chou VB, et al. Early estimates of the indirect effects of the COVID-19 pandemic on maternal and child mortality in low-income and middle-income countries: a modelling study. *Lancet Glob Health*. Jul 2020;8(7):e901-e908. doi:10.1016/s2214-109x(20)30229-1
10. Hong Xiaoa b, ∗, XDc, d, Bradley H. Wagenaar e,f, FL, OA, h,, Yan Guoi, JMUa. The impact of the COVID-19 pandemic on health services utilization in China:. *The Lancet Regional Health - Western Pacific*. March 24, 2021;9,100122, Apr 01, 2021doi:https://doi.org/10.1016/j.lanwpc.2021.100122
11. Ahmed T, Rahman AE, Amole TG, et al. The effect of COVID-19 on maternal newborn and child health (MNCH) services in Bangladesh, Nigeria and South Africa: call for a contextualised pandemic response in LMICs. *Int J Equity Health*. Mar 15 2021;20(1):77. doi:10.1186/s12939-021-01414-5
12. Wanyana D, Wong R, Hakizimana D. Rapid assessment on the utilization of maternal and child health services during COVID-19 in Rwanda. *Public Health Action*. Mar 21 2021;11(1):12-21. doi:10.5588/pha.20.0057
13. Kassie A, Wale A, Yismaw W. Impact of Coronavirus Diseases-2019 (COVID-19) on Utilization and Outcome of Reproductive, Maternal, and Newborn Health Services at Governmental Health Facilities in South West Ethiopia, 2020: Comparative Cross-Sectional Study. *Int J Womens Health*. 2021;13:479-488. doi:10.2147/ijwh.s309096
14. Singh DR, Sunuwar DR, Shah SK, et al. Impact of COVID-19 on health services utilization in Province-2 of Nepal: a qualitative study among community members and stakeholders. *BMC Health Serv Res*. Feb 24 2021;21(1):174. doi:10.1186/s12913-021-06176-y
15. Lacey A, Donna Luff. Trent Focus: Qualitative Data Analysis. 2001.
16. Contraception and COVID-19. Disrupted supply and access. 2020 April 15.
17. ICF CSACEa. Ethiopia Demographic and Health Survey 2016. Addis Ababa, Ethiopia, and Rockville, Maryland, USA: CSA and ICF2016.
18. Moreno-Montoya J BS, Rojas Sotelo JC. Impact of the COVID-19 pandemic on routine childhood immunization in Colombia, Archives of Disease in Childhood. 20 July 2021.
19. Radhika Jain a ACb, Camille Falézan a , Mustufa Patel b , Pascaline Dupas a. COVID-19 related immunization disruptions in Rajasthan, India: A retrospective observational study,. p. 4343–4350.
20. Matos CCdSA, Barbieri, C. L. A., & Couto, M. T. Covid-19 and its impact on immunization programs: reflections from Brazil.: Revista De Saúde Pública , 54 , 114; 2020.
21. Endriyas M, Alano, A., Mekonnen, E. *et al.* Understanding performance data: health management information system data accuracy in Southern Nations Nationalities and People's Region, Ethiopia.: *BMC Health Serv Res* **19,**; 2019.

22. Nigus M ZM, Abraham K, Shiferaw A, Admassu M, Masresha B. Implementing nationwide measles supplemental immunization activities in Ethiopia in the context of COVID-19: process and lessons learnt.: *Pan Afr Med J* . 2020. p. 36.
23. Moynihan R SS, Michaleff ZA, et al. Impact of COVID-19 pandemic on utilization of healthcare services: a systematic review. BMJ Open; 2021 Mar 16.
24. World health Organization (WHO) Coronavirus (COVID-19) Dashboard with vaccination data.
25. Karavadra B, Stockl, A., Prosser-Snelling, E. *et al.* Women's perceptions of COVID-19 and their healthcare experiences: a qualitative thematic analysis of a national survey of pregnant women in the United Kingdom. *BMC Pregnancy Childbirth*; 2020.
26. Goyal M SP, Singh K, Shekhar S, Agrawal N, Misra S. The effect of the COVID-19 pandemic on maternal health due to delay in seeking health care: Experience from a tertiary center.: Int J Gynaecol Obstet; 2021 Feb.
27. Bekele D, Hunegnaw BM, Bekele C, et al. Cohort profile: the Birhan Health and Demographic Surveillance System. *medRxiv*. 2021:2021.01.21.21249989. doi:10.1101/2021.01.21.21249989

Figure legends

- ✓ **Diagram 1:** Enabling (pull) factors and barriers (push factors) for service utilization highlighted in the qualitative interviews.

For peer review only - http://bmjopen.bmj.com/site/about/guidelines.xhtml

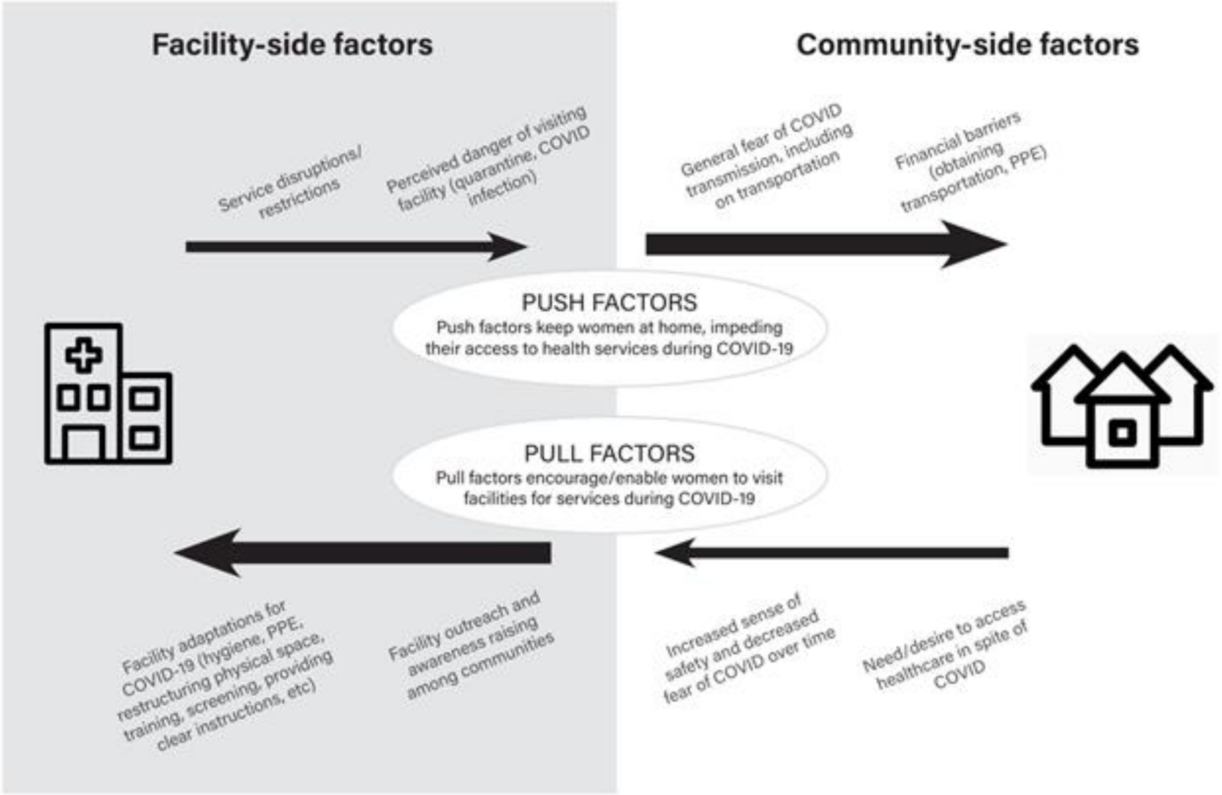
Facility-side factors
Community-side factors
Service disruptions/ restrictions
Perceived danger of visiting facility (quarantine, COVID infection)
General fear of COVID transmission, including on transportation
Financial barriers (obtaining transportation, PPE)
PUSH FACTORS
Push factors keep women at home, impeding their access to health services during COVID-19
PULL FACTORS
Pull factors encourage/enable women to visit facilities for services during COVID-19
Facility adaptations for COVID-19 (hygiene, PPE, restructuring physical space, training, screening, providing clear instructions, etc)
Facility outreach and awareness raising among communities
Increased sense of safety and decreased fear of COVID over time
Need/desire to access healthcare in spite of COVID

Supplementary Tables

Supplementary Table 1. Comparing essential MNCH service utilization over two months between COVID (April- May 2020) and analogous pre-COVID (April-May 2019) periods.

Visit Type	Mean number of visits/ months over six months		t-statistic	p-value	Lower p-value	Upper p-value	Number of paired observati
	2019	2020					
I. Maternal visit	331.3	327.3	0.05	0.959	0.520	0.480	16
1. Antenatal care	198.0	176.5	0.39	0.702	0.649	0.351	13
2. Postnatal care	17.8	20.7	-0.30	0.768	0.384	0.616	9
3. Facility delivery	85.6	87.0	-0.04	0.970	0.485	0.515	14
4. FP related services	84.1	95.1	-0.38	0.712	0.356	0.644	10
5. FP related services (hospitals, HCs and HPs combined)	289.4	227.8	0.86	0.398	0.801	0.199	16
6. Abortion-related services	10.5	10.7	-0.05	0.964	0.482	0.518	11
II. Sick child visit *(0-5years)*	201.0	126.6	1.68	0.103	0.948	0.052*	15
1. IMCI Visit (< 2 months)	7.3	3.3	1.21	0.235	0.882	0.118	15
2. IMNCI Visit (2 months – 2 year)	105.0	77.4	1.54	0.134	0.933	0.067*	15
3. IMNCI Visit (2 year – 5 year)	89.7	45.9	1.43	0.164	0.918	0.082*	15
III. Routine Immunization visit	41.0	38.1	0.16	0.875	0.563	0.437	7
1. BCG Vaccine	28.0	36.9	-0.40	0.695	0.347	0.653	10
2. Oral Polio (0) Vaccine	3.4	1.0	0.61	0.556	0.722	0.278	7
3. Pentavalent (DPT-HepB-HIP) (all types)	100.4	101.4	-0.03	0.978	0.489	0.511	10
4. Measles – 1	5.6	28.3	-1.45	0.163	0.082*	0.918	10
5. Vitamin A Dose (any dose)	6.0	1.0	0.87	0.419	0.791	0.209	4
Other types of visits							
4. All visits	2031.9	2323.5	-0.27	0.787	0.394	0.606	16
5. Adult Outpatient Visit	1811.6	2147.5	-0.29	0.773	0.386	0.614	14

* p < 0.10 ** p < 0.05 *** p < 0.01, † Lower-tailed test: mean number of visits H_1: $\mu_{2019} < \mu_{2020}$, †† Upper-tailed test: mean number of visits H_1: $\mu_{2019} > \mu_{2020}$

Supplementary Table 2. *Themes and illustrative quotes on factors enabling community facility visits during COVID-19.*

Themes	Illustrative Quotes
COVID-19 perception	
People are in doubt about COVID-19 existence in the area.	➢ I do not believe it exists, especially in our area. It might be real / exist in other areas/countries. They just suspect and take everyone into an isolation/quarantine center, but they are healthy and free of any signs and symptoms…. (W)<br>➢ ….. I have never seen anyone with such a real problem in our area. We have heard about it on radio and TV, so I found it difficult to believe and I do not believe it is real (W).<br>➢ There are huge gaps, misconceptions, and challenges in practical preventive practices. They even perceived that the disease may not be real. Clients recovered from COVID-19 without any sign and symptom disseminated the information to the community and based on that the community misconceived that the virus might not be real from the beginning (HCP).<br>➢ Right now, the entire community members have no fear or concern about acquiring the disease ……. we are not concerned about client decrement related to COVID-19. Specially after the 5 months state of emergency was lifted things are returned to pre-COVID time, …. (HCP).
No/Low COVID-19 impact perception on daily life	➢ COVID was for outsiders not for us, it was for political issues, the machine for COVID test was false (W).<br>➢ Has COVID-19 been affecting your life in any way? P: No nothing (W)<br>➢ I do not think we are at risk because we are not getting out of home most of the time and living in rural areas without any contact (W).
Knowledge on transmission methods	➢ Crowding at one place like the market and public transportation(W).<br>➢ She laughed. "Media expresses it well; we know well it is also an infected person who can transmit it …" it was not on her tip of tongue she encouraged simply to remember and told me freely "…contact, breathing" (W).<br>➢ It can be transmitted through air/ breathing, shaking hands, kissing, contact with others and when face masks are not applied properly (W).
Facility adaptation	

For peer review only - http://bmjopen.bmj.com/site/about/guidelines.xhtml

Training provided to HCP	➢ …. there was continuous and repeated awareness creation on the preventive measures, how they apply it to prevent COVID-19… (HCP)<br>➢ After the first case of COVID-19 was confirmed in our country, all health care providers including supportive staff were oriented about covid-19 and how to protect themselves and their clients (HCP).<br>➢ Training was given for all health professionals by trained woreda health professionals, how the health professional can use mask and keeping distance, source of the virus's transition and the like (HCP).
PPE use and social distancing	➢ All health workers have applied face masks and sanitizer while providing services (W).<br>➢ Health professionals kept all PPE materials in place while serving clients (HCP).<br>➢ Health professionals have put on their face masks, enforce clients to wear face masks during facility visits and hand washing soap has also been kept in place for clients (W).<br>➢ We had arranged client sitting chairs at all departments to keep their social distance; we had assigned one personnel to educate and to keep their social distance (HCP).<br>➢ We were giving care for patients face to face in and in close contact so far, but now we are providing two meters distance (HCP).

Supplementary Table 3. *Themes and illustrative quotes on perception of client flow and barriers of community facility visit.*

Themes	Illustrative Quotes
Perception of client flow	
Client flow decreased initially and increased through time	➢ During my ANC visit, I have seen some clients receiving health services. At the beginning of coronavirus some people did not want to receive the services for fear of contracting the disease. So, client flow at that time has decreased (W).<br>➢ Following of covid-19 positive case detection in the country, somewhat patient flow was decreased HCP).<br>➢ During COVID-19 time, the patient flow has dramatically decreased at the beginning (HCP).<br>➢ Right now, the entire community members have no fear or concern about acquiring the disease (HCP).<br>➢ Becomes the same as pre-COVID-19 time since the state of emergency lifted (HCP).
Barriers for service utilization	

Fair of acquiring the disease in the facility	➢ You can have this risk at transport and at health facilities during service provision and from other clients/patients. That is the first fear (HCP).<br>➢ Health professionals subjected to additional COVID-19 related tasks, patient flow decreased due to emerging concerns and fears of contracting the disease (HCP).<br>➢ I have postponed my follow up at that time for fear of acquiring the disease from health professionals/health centers. The same is true for other clients in our area and some mothers have received their visit in private clinics as we perceived almost all staff were infected (W).<br>➢ Health workers wear face masks for themselves, but they don't let all clients wear face masks during facility visits (W).
Service deprioritized	➢ As much as possible we tried to make faster service provision for their children and give advice for them not come back frequently, they can manage themselves at home if it is easy (HCP)<br>➢ We also used tele medicine for mild cases, because at the initial phase there was a direction of avoiding hospital visits for cases other than emergency (HCP).<br>➢ Initially priority was given for patients who have cough but without compromising maternal and child health care services (HCP).
Low transportation access	➢ It is also another common reason for all of us to reduce client flow to the facility (W).<br>➢ Initially …… mothers were staying at hospital unnecessarily due to absence of transportation/ambulance/ (HCP).<br>➢ In this area there was no transport restriction, but numbers were reduced to half sit and cost was doubled. It was one of the factors to reduce flow (HCP).<br>➢ Travel restrictions are also another reason for low client flow which is more pronounced among mothers from far kebeles (HCP).
Public panic	➢ At the beginning of covid-19 occurrence, the community panicked and feared acquiring the disease (HCP).<br>➢ Our basic challenge is fear of the disease. The community heard the severity of the disease in the developed country in the media, but now the problem is solved (HCP).<br>➢ Nationally the people panicked so there was a tendency of not visiting hospitals (HCP).<br>➢ The community has been frightened of contracting the disease at the beginning (W).

BMJ Open

Impact of COVID-19 Pandemic on Utilization of Facility-Based Essential Maternal and Child Health Services from March to August 2020 Compared to Pre-Pandemic March to August 2019: a Mixed Method Study in North Shewa Zone, Ethiopia

Journal:	*BMJ Open*
Manuscript ID	bmjopen-2021-059408.R1
Article Type:	Original research
Date Submitted by the Author:	09-Mar-2022

Complete List of Authors:	Bekele, Chalachew; St Paul's Hospital Millennium Medical College, Birhan HDSS<br>Bekele, Delayehu; St Paul's Hospital Millennium Medical College Department of Obstetrics and Gynecology<br>Hunegnaw, Bezawit; St Paul's Hospital Millennium Medical College, Department of Pediatrics and Child Health<br>Van Wickle, Kimiko; Harvard University T H Chan School of Public Health, Epidemiology<br>Gebremeskel, Fanos ; St Paul's Hospital Millennium Medical College, Birhan HDSS<br>Korte, Michelle ; Harvard University T H Chan School of Public Health, Department of Global Health and Population<br>Tedijanto, Christine ; University of San Francisco, Francis I. Proctor Foundation<br>Taddesse, Lisanu; Ethiopian Public Health Institute<br>Chan, Grace; Harvard Medical School, Boston Children's Hopsital, Department of Pediatrics; Harvard University T H Chan School of Public Health, Epidemiology

**Primary Subject Heading**:	Public health
Secondary Subject Heading:	Public health
Keywords:	COVID-19, Paediatric infectious disease & immunisation < PAEDIATRICS, Antenatal < GENETICS, Community child health < PAEDIATRICS, QUALITATIVE RESEARCH

SCHOLARONE™ Manuscripts

I, the Submitting Author has the right to grant and does grant on behalf of all authors of the Work (as defined in the below author licence), an exclusive licence and/or a non-exclusive licence for contributions from authors who are: i) UK Crown employees; ii) where BMJ has agreed a CC-BY licence shall apply, and/or iii) in accordance with the terms applicable for US Federal Government officers or employees acting as part of their official duties; on a worldwide, perpetual, irrevocable, royalty-free basis to BMJ Publishing Group Ltd ("BMJ") its licensees and where the relevant Journal is co-owned by BMJ to the co-owners of the Journal, to publish the Work in this journal and any other BMJ products and to exploit all rights, as set out in our licence.

The Submitting Author accepts and understands that any supply made under these terms is made by BMJ to the Submitting Author unless you are acting as an employee on behalf of your employer or a postgraduate student of an affiliated institution which is paying any applicable article publishing charge ("APC") for Open Access articles. Where the Submitting Author wishes to make the Work available on an Open Access basis (and intends to pay the relevant APC), the terms of reuse of such Open Access shall be governed by a Creative Commons licence – details of these licences and which Creative Commons licence will apply to this Work are set out in our licence referred to above.

Other than as permitted in any relevant BMJ Author's Self Archiving Policies, I confirm this Work has not been accepted for publication elsewhere, is not being considered for publication elsewhere and does not duplicate material already published. I confirm all authors consent to publication of this Work and authorise the granting of this licence.

For peer review only

Impact of COVID-19 Pandemic on Utilization of Facility-Based Essential Maternal and Child Health Services from March to August 2020 Compared to Pre-Pandemic March to August 2019: a Mixed Method Study in North Shewa Zone, Ethiopia

Authors

Chalachew Bekele, BSc MPH[1], Delayehu Bekele, MD MPH[2], Bezawit M. Hunegnaw, MD MPH[3], Kimiko Van Wickle, BSC MSPH[4], Fanos Ashenafi, BSc MSc[1], Michelle Korte, MPH[5], Christine Tedijanto, PhD[6], Lisanu Taddesse, MD MPH[7], Grace J. Chan, MD MPH PhD[3,4,8]

Affiliations

[1]Birhan HDSS, St. Paul's Hospital Millennium Medical College, Addis Ababa, Ethiopia
[2]Department of Obstetrics and Gynecology, St. Paul's Hospital Millennium Medical College, Addis Ababa, Ethiopia
[3]Department of Pediatrics and Child Health, St. Paul's Hospital Millennium Medical College, Addis Ababa, Ethiopia
[4]Department of Epidemiology, Harvard T.H. Chan School of Public Health, Boston, MA, USA
[5]Department of Global Health and Population, Harvard T.H. Chan School of Public Health, Boston, MA, USA
[6]Francis I. Proctor Foundation, University of California San Francisco, San Francisco, CA, USA
[7]HaSET MNCH research program, Addis Ababa, Ethiopia.
[8]Division of Medical Critical Care, Boston Children's Hospital, Harvard Medical School Boston, MA, USA

Corresponding Author

1. Grace J Chan, MD MPH PhD
   677 Huntington Ave, Kresge 913, Boston, MA 02115
   grace.chan@hsph.harvard.edu
2. Chalachew Bekele, BSc MPH
   Addis Ababa, Ethiopia
   cshiferaw@hsph.harvard.edu

Word Count: 4,487 (Excluding title, Abstract, figure, table and references)

Abstract word count = 296 (Excluding titles)

Abstract

Introduction: Ethiopia registered its first case of COVID-19 on March 13, 2020, and various measures were taken since then to prevent the transmission of the virus. As a result of the ongoing preventive measures and community fear of exposure, we anticipated that utilization of maternal, newborn and child health (MNCH) services at health facilities would decrease and aimed to assess the MNCH services utilization during the first six months of the COVID-19 pandemic.

Methods: The study was conducted in all BIRHAN Health and Demographic Surveillance System (HDSS) catchment health facilities in Ethiopia. Mixed study design was conducted as part of the Birhan HDSS in Ethiopia. The trend of service utilization during the first six months of COVID-19 was compared to corresponding time and data points of the preceding year.

Result: New family planning visits (43.2 to 28.5/month, p = 0.014) and sick under five child visits (225.0 to 139.8/month, P = .007) declined compared to the same period in the preceding year. Antenatal (208.9 to181.7/month, P = 0.433), and postnatal care (26.6 to 19.8/month, P = 0.155) visits, facility delivery rates (90.7 to 84.2/months, P = 0.776), aver all family planning (313.3 to 273.4/month, P = 0.415) and child routine immunization (37.0 to 36.8/month, P = 0.982) visits were maintained over the six months compared to the same period in the preceding year.

Conclusion: Provision of essential MNCH services is crucial to ascertain favorable maternal and child health outcomes. Our study presents early findings on the utilization of MNCH services that were maintained except sick child and new family planning visits. Stakeholders in low- and middle-income countries who wish to improve the resilience of their fragile health system will need to continuously monitor service utilization and clients' evolving concerns during the pandemic to prevent maternal and child morbidity and mortality.

Strengths and limitations of this study

Strengths of the study:

- ✓ We presented primary data on service utilization during the early months of the pandemic in an area of Ethiopia, one of the agrarian regions.
- ✓ The mixed methods approach integrated both quantitative service utilization coverage, and exploratory qualitative to better understand our findings and reasons for changes in service utilization.

Limitation of the study:

- ✓ We focused on coverage of service utilization as the primary outcome rather than mortality or morbidity.
- ✓ We do not have detailed data on quality-of-service provision (e.g., which services were restricted and for how long, in what manner).
- ✓ Recall bias was a possible limitation since qualitative data was collected three months later than the initial six months of the pandemic (March to August 2020).

INTRODUCTION

The World Health Organization (WHO) declared coronavirus disease-2019 (COVID-19) a global pandemic on March 11, 2020[1] and the first case of COVID-19 in Ethiopia was registered on March 13, 2020. Ethiopia was one of the countries with lower COVID-19 prevalence and related death during the study period with 63,367 confirmed cases and 974 deaths in ~119 million population as of 10 Sep 2022.[2] Majority of cases were from the city Addis Ababa and only 365 confirmed cases and 8 deaths were register up to 30 Aug 2020 in the zone (the 3rd administration unit of the country) occurred where study was conducted.

Multiple preventive measures focusing on social distancing and wearing masks were undertaken.[3] Some health facilities were assigned as COVID-19 isolation and quarantine centers, and many suspended conducting elective surgeries and select outpatient services. This increasing burden of managing COVID-19 on health facilities and health care providers leaves the health system overstretched, challenging its ability to operate effectively. As shown during the 2014-2015 Ebola outbreak in west Africa, when health systems are overwhelmed by outbreaks, mortality from vaccine-preventable and other treatable diseases can increase dramatically.[4,5]

Well-organized and prepared health systems can continue to provide equitable access to essential services throughout an emergency[6], but health systems in developing countries are often fragile when affected by emergencies such as pandemics. Accordingly, the WHO advises that countries should identify and prioritize essential services like routine vaccination, reproductive health services including care during pregnancy and childbirth, and care of young infants and older adults in their efforts to maintain continuity of service delivery and make strategic shifts to ensure that increasingly limited resources provide maximum benefit for the population.[7] The disruption of services and diversion of resources away from essential sexual and reproductive health care due to the prioritization of the COVID-19 response are expected to increase risks of maternal and child morbidity and mortality.[8]

In general lockdown or stay-at-home policy was not in place in Ethiopia where the study took place and health facilities were open during the study time period March to August 2019. The Ministry of Health (MOH) of Ethiopia focused on prevention and control of the pandemic. Possible shifts of the health workforce and the health system towards the COVID-19 response may contribute towards low utilization of routine services. For example, “half sit” policies (29 March 2020) decreased maximum occupancy on public transit, thus cutting the number of seats in half and increasing the cost of transportation when traveling within the region while transportation between regions was paused completely. Additionally, school closures (March 2020), declaration of state of emergency (08 April 2020), awareness campaigns about the pandemic, and case reports, both suspected and confirmed cases, may have all contributed to growing fear of exposure to COVID-19, especially for patients visiting health facilities.

A modelling study of essential maternal and child health interventions across 118 low- and middle-income countries over a 6-month period estimated reduction of services by 9.8–18.5% and 39.3–51.9% in the least and most severe scenarios, respectively,[9] due to the pandemic. Service reductions have already borne out in several contexts. In China, health service utilization declined

significantly after the outbreak and all indicators rebounded beginning in March, but most had not recovered to their pre-COVID-19 levels by June 2020.[10] In Bangladesh, Nigeria, and South Africa, between March and May 2020, the utilization of basic essential MNCH services such as antenatal care (ANC), family planning (FP), and immunization reduced due to lockdowns that triggered fear of contracting COVID-19, shifts of health system focused on managing the pandemic, and resource constraints.[11] During the early phase of the COVID-19 outbreak (March-April 2020) in Rwanda, utilization of ANC, deliveries, postnatal care (PNC) and immunizations significantly declined.[12] Similarly, a study in western Ethiopia showed a significant reduction in mean utilization of ANC, health facility birth, FP, and newborn immunization services between March–June 2019 and March–June 2020.[13]

In Nepal, a qualitative study found that maternity services, immunizations, and supply of essential medicine were the most affected health services during the lockdown. Interruptions were mainly due to the closure of health services at local health care facilities, limited affordability, involvement of private health sectors during the pandemic, fears of COVID-19 transmission among health care workers and within health centers, and disruption of transportation services. Participants expressed frustrations on poor testing, isolation and quarantine services related to COVID-19, and poor accountability from the government at all levels towards health services continuation and management during the COVID-19 pandemic.[14]

To understand these effects COVID-19 pandemic in Ethiopia, the HaSET maternal and child health research program assessed trends in MNCH care utilization from March 2019 to August 2020 as well as health care providers' and clients' perceptions on the barriers to and enablers of service provision and utilization during the COVID-19 emergency. This study has paramount importance in filling the evidence gap on MNCH service utilization during COVID-19, both in the Ethiopian context and other low- and middle-income countries, to prevent significant damage to the gains achieved in such areas over the past several decades.

METHODS

We conducted the study in Birhan Health and Demographic Surveillance System (HDSS) catchment health facilities in North Shewa Zone, Amhara Region, Ethiopia. The HDSS was established by Harvard University (HU) and Saint Paul's Hospital Millennium Medical College (SPHMMC) in Jun 2018, and it is a community based continuous follow up of health and demographic conditions to give up-to-date information about the catchment population and establishes a population frame to nest studies. There are five health centers, two primary hospitals (one public and one private) and one referral hospital in the area, and all (eight) catchment health facilities were selected for this study. Those facilities provide essential MNCH services for both rural (majority) and urban populations coming from HDSS catchment and non-catchment areas.

The health centers provide ANC, PNC, delivery, abortion, routine immunization (RI), integrated management of neonatal and childhood illness (IMNCI), and FP. Each health center also has a minimum of five service extension health posts mainly for FP and RI in each kebele (the simplest administration unit), and each health post sends activity reports to health centers monthly basis.

For peer review only - http://bmjopen.bmj.com/site/about/guidelines.xhtml

Two public hospitals (one primary and one referral) and the remaining one private general hospital also provide the above-mentioned essential MNCH services, except for RI, which is given mainly in health centers and catchment health posts.

Mixed phenomenological qualitative and facility-based cross-sectional study designs were employed. For the quantitative part of the study, a facility-based cross-sectional survey was conducted to assess the impact of COVID-19 on essential MNCH service provision or utilization and provider-side barriers to service provision and utilization in Birhan catchment health facilities. Ninety-one MNCH healthcare providers (doctors, nurses, midwives, and clinical officers available at the time of data collection) were asked with uniformly structured questionnaires about their perception of client flow and possible barriers for respective sections. Twelve out of 91 healthcare providers were working in two MNCH departments and interviewed twice. In addition to this, healthcare utilization time-series data from each facility was retrospectively collected from medical records and monthly facility reports. Retrospective facilities service statistics were collected over an 18-month period from March 2019 to August 2020 using Computer Assisted Field Editing (CAFE). Data was abstracted by uniformly structured questionnaires and entered to Open Data kit (ODK) collect and uploaded to ODK aggregate. The facilities' monthly reports and medical registers data were collected separately. The health centers monthly reports include services given in the health posts that are extension sites for the health center, but the facility registers are exclusively for services given in the health centers.

In addition to the cross-sectional study, a phenomenological qualitative design utilizing in-depth interviews was implemented to assess client and provider side barriers and enablers to service provision/utilization in Birhan catchment health facilities. Purposive sampling was implemented, and in-depth interviews were conducted until theoretical saturation was reached. Ten facility or department heads, and nine women (delivered at home/facility, had ANC, or missed ANC follow up) were interviewed. An interview guide with open-ended questions was translated from English to Amharic and used to elicit the qualitative information from informants and face-to-face interviews were conducted in the facilities with facility/department heads, women who visited facilities during COVID-19 and women who delivered at home. Women who missed an ANC follow up were interviewed by phone. With the permission of the respondent, all interviews were recorded, and all recorded data was transcribed to English for further analysis. To ensure the safety of the data collectors and participants, masks were worn, and social distancing practices were implemented during training and data collection from 2 – 20 November 2020.

The extracted data was exported to Stata 17.0 for analysis and the average MNCH services uptake was calculated each month to quantify the changes pre – COVID-19 (March to August 2019) and during the COVID-19 (March to August 2020) pandemic. For the purposes of analysis, March to August 2019 and March to August 2020 were considered as pre-COVID-19 and COVID-19 periods, respectively. We had 48 paired months observations of essential MNCH variables except RI, which was 30 paired months, since it was given only in five health centers including extension health posts. The facilities medical records were archived misplaced, and we found some months data missing and partially field. To avoid the effect of missing and partially filled values, analogous months data from the same facility were excluded from the data analysis. Finally, an independent sample t-test was done to compare pre-COVID-19 and COVID-19 time

months. This analysis was repeated for the initial two-months (March to April 2020) of the pandemic and the analogous period, March to April 2019, to examine changes in service utilization at the onset of the COVID-19 pandemic and a significance level of $\alpha=0.05$ was used for all statistical tests.

In addition to the quantitative metrics listed above, English language transcript data was entered in Dedoose software for qualitative data analysis. After familiarization with the data, the content of the data was coded line by line for thematic analysis following a framework theory approach to describe and interpret health providers' and communities' perceptions on barriers and enablers to MNCH service provision. The framework approach involves using some pre-assigned themes to initially categorize data while also adjusting and iterating the coding scheme to accommodate newly emergent themes, sub-themes, and categories through inductive interpretation.[15] Coded data was examined for potential relationships and themes were also assessed across relevant participant demographic categories to understand different user perspectives. Findings were described under pre-assigned and newly emerged themes.

Ethic Statement

This study involves human participants and was approved by Ethics Review Board (IRB) of Saint Paul's Hospital Millennium Medical college (SPHMMC) and Harvard T.H. Chan School of Public Health (HSPH) (IRB20-1574). To extract MNCH service statistics, permission was obtained from individual health facilities and individual verbal consent was obtained from respondents.

Patient and Public Involvement

Meeting was restricted during protocol development and study period due to COVID-19 pandemic and it was not possible to involve clients or the public in the design, study, reporting and dissemination plans of our research.

RESULTS

For the quantitative section of the study, data were abstracted from a total of eight health facilities (three hospitals and five health centers) and interviews with 103 healthcare providers working in the MNCH units of the facilities. In addition to these, ten facility or MNCH department heads and nine women (pregnant and delivered in the time of COVID-19) were asked open-ended questions.

We did not see a statistically significant change in maternal health facility visits for ANC, PNC, facility delivery, and abortion related. The FP services utilization in the health centers and hospitals declined from 105.5 to 66.5 visits per month ($p < 0.05$) after the onset of the pandemic and within the subset of FP visits, repeat and unclassified FP visits significantly declined while new FP visits did not change. Since the FP services are given in health posts, the source of data for FP is aggregated data from both health posts and health centers[16] and the overall FP service utilization was maintained (313.3 to 273.4/month, $P = 0.415$), except, the new FP services which declined significantly from 43.2 visits per month to 28.5 visits per month ($p = 0.029$).

Declines in service utilization were found among sick child visits, which was defined as a facility visit for sick children under five years old. The mean number of IMNCI visits for sick children under 5 years old declined from 225.0 visits per month in 2019 to 139.8 visits per month in 2020 (p = 0.014). This significant relationship persists for two age stratifications of IMNCI visits (2 months to under 2 years, and 2 years to under 5 years). On the other hand, there was no significant change (37.0 to 36.8/month, P = 0.982**)** in child visits for routine immunizations, including BCG, OPV-0, pentavalent (DPT-HepB-HIP) and measles vaccinations **(Table 1).**

Ninety-one healthcare providers who were working in maternal, newborn and child health were asked about the client flow during COVID-19. Sixty-seven percent of the health care providers (HCPs) perceived that client flow decreased and 31% of them considered the same **(Supp Table 1)**. Qualitative interviews also supported the observed decrease in client flow, with descriptions of sharper contractions in service utilization in the first couple of months after the onset of the pandemic but resumed to approximately normal levels over subsequent months. To explore the perception of lower service utilization during the initial couple of months of COVID-19 by clients of the health system and HCPs, data on the initial two pre-COVID (March to April 2019) months were compared with analogous COVID time months (March to April 2020) and there were no statistically significant changes in the number of visits for maternal and childhood visits overall, except sick child visit **(Supp Table 2)**.

Barriers to service provision and utilization during COVID-19

Even though the essential MNCH service utilization was maintained; clients' fear of acquiring the disease from the facility, travel restrictions, increased transportation cost due to the half seat order by the government, and fear of acquiring the disease on the way to the health facility were the main barriers for service utilization perceived by healthcare providers **(Table 2).**

These secondhand perspectives from healthcare providers about the barriers that clients face was largely supported by qualitative interviews with clients. Fear of contracting the disease and lack of access to transportation are the most described barriers **(Supp table 3).** Particularly during the first few months after the onset of COVID-19 in Ethiopia and the imposition of travel restrictions and other public health measures like state of emergency, communities fear of acquiring the disease and high levels of public panic were barriers for facility-based services utilization. This fear extended to visiting facilities during the pandemic; community members were afraid of contracting the disease in crowded spaces, public transportation routes to facilities, or at facilities themselves from health care workers or other patients, particularly when they heard of COVID-19 cases present at facilities and this fear often resulted in delayed care-seeking. A woman said that *"I have postponed my follow up at that time for fear of acquiring the disease from health professionals and health centers. The same is true for other clients in our area, and some mothers have received their visit in private clinics as we perceived almost all staff were infected"*.

The economic hardship during COVID-19 prevented some clients from being able to pay for transportation due to transportation restriction with half seats and doubled transportation fee, and other direct or indirect costs of attending facilities. Clients described lacking money to purchase

PPE and one HCP noted that the closed market movement affected people's incomes, as reflected by patients delaying treatment until conditions are more severe or defaulting on treatment.

Lastly, bottlenecks on the health system side provided another barrier to service utilization. Facilities restricted some services at the beginning of the pandemic's onset and clients were unable to access certain services or assumed that services were restricted even after they had resumed, as one HCP suggested. Additionally, multiple clients described fearing that they might be forcibly quarantined or presumed COVID-19-positive if they were to visit facilities and this fear deterred facility visits. HCPs described many challenges related to the under preparedness of the health system for managing suspected cases of COVID-19. Often these challenges manifested through physical infrastructure constraints and a shortage of guidelines for managing quarantine and isolation centers for suspected COVID-19 cases.

Enablers of service provision and utilization during COVID-19

In terms of knowledge of COVID-19, all women had heard about the disease, but a few were in doubt about COVID-19 existence in the area which may be an enabling factor for facility-based service utilization. A client respondent said, "*I do not believe it exists, especially in our area. It might be real / exist in other areas/countries. They just suspect and take everyone into an isolation/quarantine center, but they are healthy and free of any signs and symptoms…*". Some described that COVID-19 could not affect them because God and/or Mary will protect them, citing the importance of prayer as a protective measure.

Facility adaptations like training for healthcare providers, hand washing facilities, physical distancing, and awareness creation and health education which was given by local authorities increased the client awareness on COVID-19 prevention and facility-based service utilization through time **(Supp table 4).**

Understanding Service Utilization Trends during COVID-19

The barriers and enablers highlighted in the interviews interact with each other dynamically, as depicted below. Certain barriers were more substantial than others, particularly fear of the disease, transportation access, and economic-related barriers, while certain pull factors encouraged facility visits, particularly over time as fear subsided, community awareness measures were undertaken, and facilities implemented adaptations to manage both COVID-19 and routine services **(Diagram 01).**

DISCUSSION

We examined the impact of the COVID-19 pandemic on essential MNCH service utilization by analyzing data from health facility records and healthcare providers and patients' perspectives. The MNCH services utilization were maintained over six months compared to the same period in the preceding year, except new family planning initiation and sick under five years old child visits. Although our samples were eight health facilities available in the HDSS they are likely to

be representative because clients and healthcare providers' views were added in addition to six analogous months data abstraction from medical records.

Studies reported a 37% overall healthcare utilization and 42% of visit reduction[17] and ANC, PNC and facility delivery were affected in African coutries.[11,12,18,19] On the other hand FP, institutional delivery, RI and ANC did not vary significantly between pre-COVID-19 and during COVID-19 in Amhara region, Ethiopia[20] and Kenya. [21] Similarly, our study showed that maternal facility-based healthcare provision was maintained and it might be due to the government response for COVID-19, because the stay at home/lockdown policy was not in place, the prevalence of confirmed cases and death were lower, and facilities were open for MNCH services through study period.

Globally, contraception services were shut down or not accessible [22] and the service provision was declined. [23,24] In our study health center and hospital based family planning service utilization decreased (105.5 to 66.5/ months, P = 0.051), but the family planning service is given by health extension workers at health posts which are service extension sites of health centers in the villages and the combined FP service utilization was maintained (313.3 to 273.4/month, P = 0.415), except new FP initiations which was significantly affected (43.2 to 28.5/month, P = 0.029). This is suggesting that utilization of family planning services was more likely to occur when uncrowded clinics are nearby without extensive travel.

For child health, the number of Integrated Management of Neonatal and Childhood Illness (IMNCI) visits, also referred to as sick child visits, significantly declined by 38% (**225.0 to 139.8, P = 0.014**). Similarly, child health service declined by 33% in three sub-Saharan Africa countries including Ethiopia due to COVID-19[24] and it is possible that the decrease in child sick visits was related to COVID-19 prevention and control activities. The leading causes of under five years old children morbidity in Ethiopia,[25] acute respiratory illness, fever and diarrhea may have decreased due to school closures (older siblings less exposed), limited interactions with peers in the community, spending more time indoors, mask-wearing at community gatherings, hand washing, physical distancing, and other personal protective equipment and practices. We found that RI remained stable during the initial six months of the pandemic, which was different than the findings from studies in Colombia, India, and Brazil where RI declined during the pandemic.[26-28] The MOH of Ethiopian prioritized RI, especially measles, during COVID-19 and existing health extension workers stationed at health posts for community-based services hygiene and sanitation, FP and RI which may have sustained accessibility to these services during COVID-19 because home to home visits creates close relations with clients and health posts are not crowded., even though, there was a marked reduction in supply chain distribution of vaccines in Ethiopia during COVID-19.[29]

At the time of data collection, early in the pandemic, respondents largely described not feeling many tangible impacts of COVID-19 on their daily lives if they fail to adherence to preventive measures, so they went about life as usual. This easing fear of COVID-19 may have enabled women to feel that they could safely attend services, but it also has important implications as the pandemic continues, particularly as cases in Ethiopia have risen substantially. Awareness and education campaigns are needed to produce actual behavior change. Moreover, communities' belief that God may protect them from infection indicates the important role of engaging religious

For peer review only - http://bmjopen.bmj.com/site/about/guidelines.xhtml

leaders as champions in behavior change campaigns. An additional key recommendation is systematically addressing misinformation and doubt to increase population compliance with preventive measures, particularly as Ethiopia faces a rising caseload, increasing prevalence of variants, and a stalled vaccine rollout that may take months or years to reach substantial population coverage. Less than 8.4% of the population has received at least one dose of COVID-19 vaccine as of 19 Feb 2022.[30]

Barriers to maternal facility visits included women not wanting to bother anyone, lack of support from healthcare workers, influence of the media,[31] lockdown, fear of contracting the disease,[32] shift of focus towards pandemic, resource constraints[11] and non-conducive working environments for healthcare providers[33]. In addition, women experienced fears of contracting the disease, economic hardship, and lack of access to transportation. Particularly during the first few months after the onset of COVID-19 in Ethiopia, there was an imposition of travel restrictions and other public health measures like state of emergency, and high levels of public panic. Multiple clients described fearing that they might be forcibly quarantined or presumed COVID-19-positive if they were to visit facilities; this fear deterred facility visits. While we found that sick child visits and new family planning services were affected by the pandemic, the declines among other essential services were not as significant indicating hope in service resilience and the ability to introduce rapid and substantial facility adaptations to maintain the health system (e.g., personal protective equipment, infection prevention measures, improved sanitation/hygiene).

Strengths of the study: We present primary data on service utilization during the early months of the pandemic in an area of Ethiopia, one of the agrarian regions, which is generalizable to 80% of the country's rural population. [34] We leveraged an existing research network, HaSET MNCH research program (www.hasetmch.org), and established field site[35] to rapidly collect data from all available sources. The mixed methods approach integrated both quantitative service utilization coverage data with sociocultural, contextual, and exploratory qualitative to better understand our findings and reasons for changes in service utilization. The study highlights success stories in community-based care and government leadership for key services like routine immunization that may benefit other settings.

Limitation of the study: Our study focused on service utilization and may not have been powered to detect significant differences. Furthermore, In Kenya, Tanzania and Uganda, maternal and neonatal mortality rates exacerbated due to COVID-19, [36] but we couldn't see this effect in the Ethiopian context since we focused on coverage of service utilization as the primary outcome rather than mortality or morbidity. We do not have detailed data on service provision (e.g., which services were restricted and for how long, in what manner). There is the potential of recall bias were possible limitation since qualitative data was collected three months later than the initial six months of the pandemic (March to August 2020).

CONCLUSION

Utilization of essential MNCH services is crucial to achieve favorable health outcomes. In the setting of developing countries like Ethiopia, health systems are often too fragile to withstand the direct increase in volume of patients and indirect health consequences of a pandemic. Our study presents early findings of maintained utilization of MNCH services, except new family planning

For peer review only - http://bmjopen.bmj.com/site/about/guidelines.xhtml

services and sick child visits. Further study is needed to assess the effect of the pandemic on women and children morbidity and mortality. To prevent worsening maternal and child morbidity and mortality as a result of the pandemic, resources are required by government leaders, policy makers, and clinicians to improve the resilience of their health system to continuously monitor service utilization, while at the same time engaging with providers and clients to understand and address their evolving concerns about MNCH service uptake.

ABBREVIATIONS

CAFÉ: Computer Assisted Field Editing, COVID-19: Coronavirus Disease - 2019, DPT: Diphtheria Pertussis and Tetanus, EOC: Emergency Operation center, EPHI: Ethiopian Public Health Institute, FP: Family Planning, HC: Health Center, HCP: Healthcare provider, HDSS: Health and Demographic Surveillance System, HepB: Hepatitis B, HIP: Haemophilus influenzae, HIV/AIDS: Human Immunodeficiency Virus/ Acquired Immune Deficiency Syndrome, HP: Health post, HSPH: Harvard School of Public Health, IRB: Institutional Review Board, MCH: Maternal and Child Health, MNCH: Maternal, Newborn and Child Health, MOH: Ministry of Health, ODK: Open Data Kit, PNC: Postnatal care, RI: Routine immunization, RMNCH: Reproductive, Maternal, Newborn and Children Health, SPHMMC: Saint Paul Hospital Millennium Medical College, W: Women, WHO: World Health organization.

ACKNOWLEDGEMENTS

We acknowledge the kind help and encouragement we received from the zone, woreda and study facilities. Our deepest gratitude goes to the women and health care providers who participated in this study and to all data collectors and supervisors for their dedicated work.

FUNDING

This work is supported by the Bill & Melinda Gates Foundation.

GRANT/AWARD NUMBER

The research is co-funded through INV-006752 HaSET and INV-003612 ANC/PNC Innovations Platform HaSET Ethiopia Partnership.

AVAILABILITY OF DATA AND MATERIALS

The datasets used and/or analyzed during the current study are available from the corresponding author on reasonable request.

COMPETING INTERESTS

The authors declare that they have no competing interests.

References

1. World, Organization H. Coronavirus disease (COVID-19) situation report – 162. 2020.
2. https://www.worldometers.info/coronavirus/.

For peer review only - http://bmjopen.bmj.com/site/about/guidelines.xhtml

3. Agency EP. Ethiopia officially declares State of Emergency. April 9, 2020. https://www.press.et/english/p=20750.
4. Elston JW, Cartwright C, Ndumbi P, Wright J. The health impact of the 2014-15 Ebola outbreak. Public Health. Feb 2017; 143:60-70. Doi: 10.1016/j.puhe.2016.10.020.
5. Parpia AS, Ndeffo-Mbah ML, Wenzel NS, Galvani AP. Effects of Response to 2014-2015 Ebola Outbreak on Deaths from Malaria, HIV/AIDS, and Tuberculosis, West Africa. Emerg Infect Dis. Mar 2016; 22(3): 433-41. Doi:10.3201/eid2203.150977.
6. Organization WH. 2019 novel coronavirus (2019‑nCoV): strategic preparedness and response plan. 20 February 2020.
7. Organization WH. WHO releases guidelines to help countries maintain essential health services during the COVID-19 pandemic. March 30, 2020.
8. Hall KS, Samari G, Garbers S, et al. Centring sexual and reproductive health and justice in the global COVID-19 response. Lancet. Apr 11, 2020; 395(10231):1175-1177. Doi:10.1016/s0140-6736(20)30801-1.
9. Roberton T, Carter ED, Chou VB, et al. Early estimates of the indirect effects of the COVID-19 pandemic on maternal and child mortality in low-income and middle-income countries: a modelling study. Lancet Glob Health. Jul 2020;8(7): e901-e908. Doi:10.1016/s2214-109x(20)30229-1.
10. Hong Xiaoa b, XDc, d, Bradley H. Wagenaar e,f, FL, OA, h,, Yan Guoi, JMUa. The impact of the COVID-19 pandemic on health services utilization in China: The Lancet Regional Health - Western Pacific. March 24, 2021;9,100122, Apr 01, 2021. Doi: https://doi.org/10.1016/j.lanwpc.2021.100122
11. Ahmed T, Rahman AE, Amole TG, et al. The effect of COVID-19 on maternal newborn and child health (MNCH) services in Bangladesh, Nigeria and South Africa: call for a contextualised pandemic response in LMICs. Int J Equity Health. Mar 15, 2021; 20(1):77. Doi:10.1186/s12939-021-01414-5
12. Wanyana D, Wong R, Hakizimana D. Rapid assessment on the utilization of maternal and child health services during COVID-19 in Rwanda. Public Health Action. Mar 21, 2021;11(1):12-21. Doi:10.5588/pha.20.0057
13. Kassie A, Wale A, Yismaw W. Impact of Coronavirus Diseases-2019 (COVID-19) on Utilization and Outcome of Reproductive, Maternal, and Newborn Health Services at Governmental Health Facilities in Southwest Ethiopia, 2020: Comparative Cross-Sectional Study. Int J Womens Health. 2021; 13:479-488. Doi:10.2147/ijwh.s309096

For peer review only - http://bmjopen.bmj.com/site/about/guidelines.xhtml

14. Singh DR, Sunuwar DR, Shah SK, et al. Impact of COVID-19 on health services utilization in Province-2 of Nepal: a qualitative study among community members and stakeholders. BMC Health Serv Res. Feb 24, 2021;21(1):174. Doi:10.1186/s12913-021-06176-y
15. Lacey A, Donna Luff. Trent Focus: Qualitative Data Analysis. 2001.
16. Endriyas M, Alano, A., Mekonnen, E. et al. Understanding performance data: health management information system data accuracy in Southern Nations Nationalities and People's Region, Ethiopia.: BMC Health Serv Res 19, 2019.
17. Moynihan R SS, Michaleff ZA, et al. Impact of COVID-19 pandemic on utilization of healthcare services: a systematic review. BMJ Open; 2021 Mar 16.
18. Ameyaw EK, Ahinkorah, B.O., Seidu, AA. et al. Impact of COVID-19 on maternal healthcare in Africa and the way forward. 201;223 (2021). Doi: https://doi.org/10.1186/s13690-021-00746-6
19. Burt JF OJ, Lubyayi L ea. Indirect effects of COVID-19 on maternal, neonatal, child, sexual and reproductive health services in Kampala, Uganda. BMJ Global Health. 2021;6: e006102. Doi:10.1136/ bmjgh-2021-006102
20. Wendemagegn Enbiale SGA, Meaza Seyum, Dereje Bedanie Hundie, Kassawmar Angaw Bogale, Koku Sisay Tamirat, Mulat Birhanu Feleke, Muluken Azage, Dabere Nigatu, and Henry J. C. de Vries. Effect of the COVID-19 Pandemic Preparation and Response on Essential Health Services in Primary and Tertiary Healthcare Settings of Amhara Region, Ethiopia. Am J Trop Med Hyg. 2021;105(5):1240–1246. Doi:10.4269/ajtmh.21-0354
21. Shikuku1 D, IN, SG, et al. Early indirect impact of COVID-19 pandemic on utilization and outcomes of reproductive, maternal, newborn, child, and adolescent health services in Kenya. medRxiv; 2021.
22. Contraception and COVID-19. Disrupted supply and access. 2020 April 15.
23. Paulo Henrique das Neves Martins Pires CM, Ahmed Abdirazak, Jaibo Rassul Mucufo, Martins Abudo Mupueleque DZ, Ronald Siemens and Celso Fernando Belo. Covid-19 pandemic impact on maternal and child health services access in Nampula, Mozambique: mixed methods research. BMC Health Serv Res 21, 860 (2021). Doi: https://doi.org/10.1186/s12913-021-06878-3
24. Nega Assefa AS, Dongqing Wang, Michelle L. Korte, Elena C. Hemler, Yasir Y. Abdullahi, Bruno Lankoande, Ourohire Millogo, Angela Chukwu, Firehiwot Workneh, Phyllis Kanki, Till Baernighausen, Yemane Berhane, Wafaie W. Fawzi,and Ayoade Oduola. Reported Barriers to Healthcare Access and Service Disruptions Caused by COVID-19 in Burkina

For peer review only - http://bmjopen.bmj.com/site/about/guidelines.xhtml

Faso, Ethiopia, and Nigeria: A Telephone Survey. Am J Trop Med Hyg. 2021;105(2):323–330. Doi:10.4269/ajtmh.20-1619

25. ICF CSACEa. Ethiopia Demographic and Health Survey 2016. Addis Ababa, Ethiopia, and Rockville, Maryland, USA: CSA and ICF2016.
26. Moreno-Montoya J BS, Rojas Sotelo JC. Impact of the COVID-19 pandemic on routine childhood immunization in Colombia, Archives of Disease in Childhood. 20 July 2021.
27. Radhika Jain a ACb, Camille Falézan a, Mustufa Patel b, Pascaline Dupas a. COVID-19 related immunization disruptions in Rajasthan, India: A retrospective observational study,. p. 4343–4350.
28. Matos CCdSA, Barbieri, C. L. A., & Couto, M. T. Covid-19 and its impact on immunization programs: reflections from Brazil.: Revista De Saúde Pública , 54 , 114; 2020.
29. Nigus M ZM, Abraham K, Shiferaw A, Admassu M, Masresha B. Implementing nationwide measles supplemental immunization activities in Ethiopia in the context of COVID-19: process and lessons learnt.: Pan Afr Med J. 2020. p. 36.
30. World health Organization (WHO) Coronavirus (COVID-19) Dashboard with vaccination data.
31. Karavadra B, Stockl, A., Prosser-Snelling, E. et al. Women's perceptions of COVID-19 and their healthcare experiences: a qualitative thematic analysis of a national survey of pregnant women in the United Kingdom. BMC Pregnancy Childbirth; 2020.
32. Goyal M SP, Singh K, Shekhar S, Agrawal N, Misra S. The effect of the COVID-19 pandemic on maternal health due to delay in seeking health care: Experience from a tertiary center.: Int J Gynaecol Obstet; 2021 Feb.
33. Kayiga H GD, Amuge PM, Ssemata AS, Nanzira RS, Nakimuli A. Lived experiences of frontline healthcare providers offering maternal and newborn services amidst the novel corona virus disease 19 pandemic in Uganda: A qualitative study.: PLoS One; 10 Dec 2021.
34. Ethiopia Demographics 2020 (Population, Age, Sex, Trends) - Worldometer (worldometers.info).
35. Bekele D, Hunegnaw BM, Bekele C, et al. Cohort profile: the Birhan Health and Demographic Surveillance System. medRxiv. 2021:2021.01.21.21249989. doi:10.1101/2021.01.21.21249989
36. Eunice Pallangyo MGN, Rose Maina, Valerie Fleming, The impact of covid-19 on midwives' practice in Kenya, Uganda, and Tanzania: A reflective account, Midwifery. 2020;89(0266-6138). Doi: https://doi.org/10.1016/j.midw.2020.102775.

Figure and tables

Diagram 1: Enabling (pull) factors and barriers (push factors) for service utilization highlighted in the qualitative interview

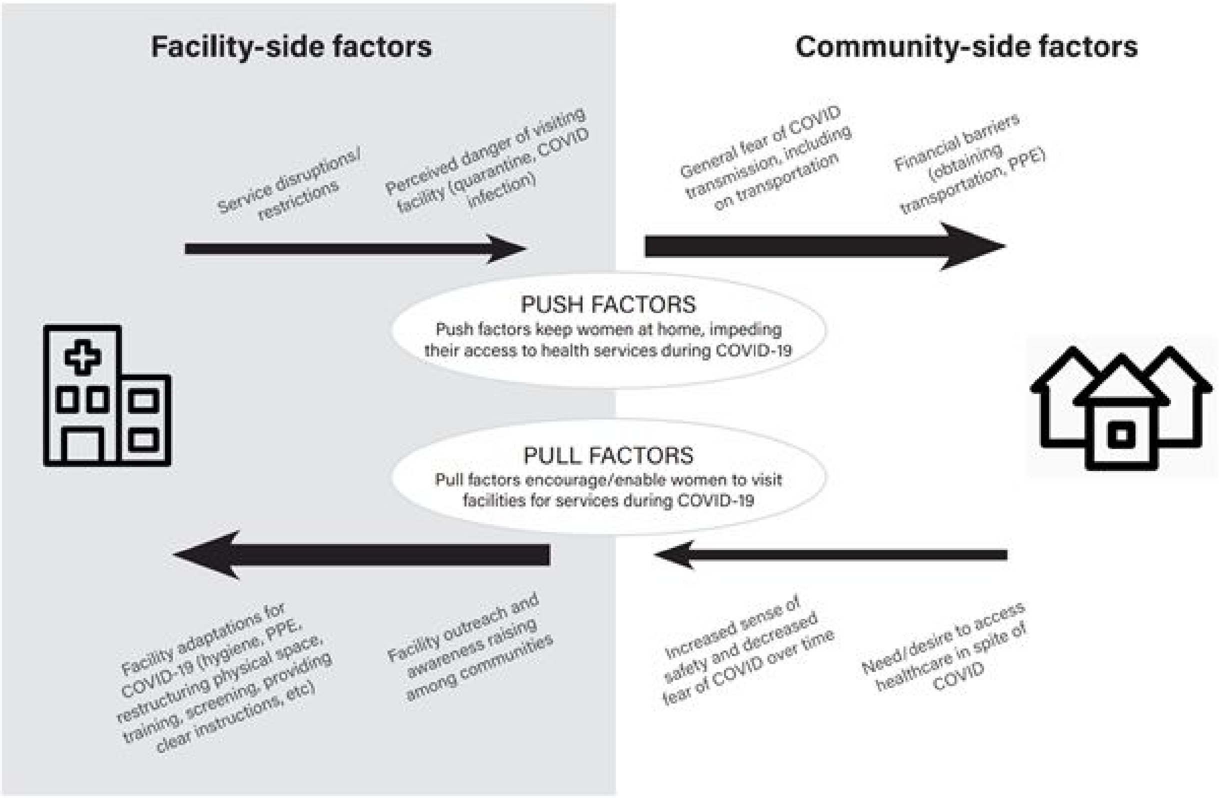

Table 1. *Comparing essential MNCH service utilization over six months between COVID-19 (Mar-Aug 2020) and analogous pre-COVID-19 (Mar-Aug 2019) periods.*

Visit Type	Mean number of visits/month over six months		t-statistic	p-value	Lower p-value †	Upper p-value ††	Paired observations
	2019	2020					
**I. Maternal visit**	**376.3**	**321.2**	**1.11**	**0.270**	**0.865**	**0.135**	**48/48**
1. Antenatal care	208.9	181.7	0.79	0.433	0.784	0.216	40/48
2. Postnatal care	26.6	19.8	1.44	0.155	0.922	0.078*	30/48
3. Facility delivery	90.7	84.2	0.29	0.776	0.612	0.388	41/48

4. Abortion-related services	11.8	9.8	0.56	0.578	0.711	0.289	34/48
5. Overall FP services in health posts, health centers and hospitals	313.3	273.4	0.82	0.415	0.792	0.207	47/48
5.1 New FP services	43.2	28.5	1.22	0.029	0.986	0.014**	47/48
5.2 Repeat FP services	270.2	244.9	0.57	0.567	0.716	0.284	47/48
6. FP services in health centers and hospitals	105.5	66.5	1.99	0.051	0.974	0.026**	33/48
6.1 New FP services	8.9	7.1	0.84	0.406	0.797	0.203	33/48
6.2 Repeat FP services	96.5	59.3	1.03	0.046	0.977	0.023**	33/48
6.3 Unclassified FP services	17.7	1.3	1.12	0.039	0.981	0.019**	26/48
**II. Sick child visit (0-5years))**	**225.0**	**139.8**	**1.51**	**0.014**	**0.993**	**0.007*****	46/48
1. MNCI Visit (< 2 months)	10.8	7.7	0.82	0.412	0.794	0.206	46/48
2. IMNCI Visit (2 months – 2 year)	101.6	50.4	1.68	0.009	0.996	0.004***	46/48
3. IMNCI Visit (2 year – 5 year)	111.6	81.8	1.15	0.034	0.983	0.017**	46/48
**III. Routine Immunization visit**	**37.0**	**36.8**	**0.02**	**0.982**	**0.509**	**0.491**	23/30
1. BCG Vaccine	31.4	36.5	-0.39	0.701	0.350	0.650	30/30
2. Oral Polio (0) Vaccine	3.2	1.0	0.88	0.384	0.808	0.192	23/30
3. Pentavalent (DPT-HepB-HIP) (all types)	100.4	101.5	-0.05	0.958	0.479	0.521	30/30
4. Measles – 1	10.3	27.5	-1.99	0.051	0.026	0.974	30/30
5. Vitamin A Dose	8.6	5.7	0.67	0.506	0.747	0.253	14/30
**Other types of visits**							

For peer review only - http://bmjopen.bmj.com/site/about/guidelines.xhtml

All visits	2568.9	2606.7	-0.05	0.956	0.478	0.522	48/48
Adult Outpatient Visit	2121.2	2239.7	-0.17	0.868	0.434	0.566	44/48
* $p < 0.10$ ** $p < 0.05$ *** $p < 0.01$, † Lower-tailed test: mean number of visits H_1: $\mu_{2019} < \mu_{2020}$, †† Upper-tailed test: mean number of visits H_1: $\mu_{2019} > \mu_{2020}$							

Table 2. *Possible barriers to service utilization in the time of COVID-19 based on healthcare providers' perception.*

Possible barriers to service utilization in the time of COVID-19	Count	%
1. Fear of acquiring the diseases from the facility	97	94
2. Travel restrictions	90	87
3. Increased transportation cost (due to half sit order by the government)	89	86
4. Fear of acquiring the disease on the way to the health facility	86	83
5. Lack of transport to the HP/HC site	72	70
6. Lack of PPE for clients	67	65
7. Clients' perception of limited implementation of protective measures by healthcare providers	58	56
8. Healthcare providers advice to stay at home	54	52
9. Limited-service hours or absence of health care workers	17	17
10. Unavailability of ambulance	7	7
11. Unavailability of healthcare providers in facilities to provide outreach services.	7	7
Total interviews	**103**	

Note: *Twelve healthcare providers were working in different departments and asked twice.*

For peer review only - http://bmjopen.bmj.com/site/about/guidelines.xhtml

Supplementary Tables

Supplementary Table 1: Healthcare providers' perception about MNCH clients flow to the facility in the time of COVID-19.

Response	Count	Percent
Increasing	2	2%
The same	28	31%
Decreasing	61	67%
Total	91	100%

Supplementary Table 2. Comparing essential MNCH service utilization over two months between COVID (April- May 2020) and analogous pre-COVID (April-May 2019) periods.

Visit Type	Mean number of visits/ months over six months		t-statistic	p-value	Lower p-value	Upper p-value	Number of paired observations
	2019	2020					
I. Maternal visit	331.3	327.3	0.05	0.959	0.520	0.480	16
1. Antenatal care	198.0	176.5	0.39	0.702	0.649	0.351	13
2. Postnatal care	17.8	20.7	-0.30	0.768	0.384	0.616	9
3. Facility delivery	85.6	87.0	-0.04	0.970	0.485	0.515	14
4. FP related services	84.1	95.1	-0.38	0.712	0.356	0.644	10
5. FP related services (hospitals, HCs and HPs combined)	289.4	227.8	0.86	0.398	0.801	0.199	16
6. Abortion-related services	10.5	10.7	-0.05	0.964	0.482	0.518	11
II. Sick child visit *(0-5years)*	201.0	126.6	1.68	0.103	0.948	0.052*	15

1. IMCI Visit (< 2 months)	7.3	3.3	1.21	0.235	0.882	0.118	15
2. IMNCI Visit (2 months – 2 year)	105.0	77.4	1.54	0.134	0.933	0.067*	15
3. IMNCI Visit (2 year – 5 year)	89.7	45.9	1.43	0.164	0.918	0.082*	15
III. Routine Immunization visit	41.0	38.1	0.16	0.875	0.563	0.437	7
1. BCG Vaccine	28.0	36.9	-0.40	0.695	0.347	0.653	10
2. Oral Polio (0) Vaccine	3.4	1.0	0.61	0.556	0.722	0.278	7
3. Pentavalent (DPT-HepB-HIP) (all types)	100.4	101.4	-0.03	0.978	0.489	0.511	10
4. Measles – 1	5.6	28.3	-1.45	0.163	0.082*	0.918	10
5. Vitamin A Dose (any dose)	6.0	1.0	0.87	0.419	0.791	0.209	4
Other types of visits							
4. All visits	2031.9	2323.5	-0.27	0.787	0.394	0.606	16
5. Adult Outpatient Visit	1811.6	2147.5	-0.29	0.773	0.386	0.614	14

* p < 0.10 ** p < 0.05 *** p < 0.01, † Lower-tailed test: mean number of visits H_1: $\mu_{2019} < \mu_{2020}$, †† Upper-tailed test: mean number of visits H_1: $\mu_{2019} > \mu_{2020}$

Supplementary Table 3. *Themes (authors interpretation) and illustrative quotes of key informants on factors enabling community facility visits during COVID-19.*

Themes	Illustrative Quotes
COVID-19 perception	
People are in doubt about COVID-19	➢ I do not believe it exists, especially in our area. It might be real / exist in other areas/countries. They just suspect and take everyone into an isolation/quarantine center, but they are healthy and free of any signs and symptoms…. (W)

existence in the area.	➢ ….. I have never seen anyone with such a real problem in our area. We have heard about it on radio and TV, so I found it difficult to believe and I do not believe it is real (W).<br>➢ There are huge gaps, misconceptions, and challenges in practical preventive practices. They even perceived that the disease may not be real. Clients recovered from COVID-19 without any sign and symptom disseminated the information to the community and based on that the community misconceived that the virus might not be real from the beginning (HCP).<br>➢ Right now, the entire community members have no fear or concern about acquiring the disease ……. we are not concerned about client decrement related to COVID-19. Specially after the 5 months state of emergency was lifted things are returned to pre-COVID time, …. (HCP).
No/Low COVID-19 impact perception on daily life	➢ COVID was for outsiders not for us, it was for political issues, the machine for COVID test was false (W).<br>➢ Has COVID-19 been affecting your life in any way? P: No nothing (W)<br>➢ I do not think we are at risk because we are not getting out of home most of the time and living in rural areas without any contact (W).
Knowledge on transmission methods	➢ Crowding at one place like the market and public transportation(W).<br>➢ She laughed. "Media expresses it well; we know well it is also an infected person who can transmit it …" it was not on her tip of tongue she encouraged simply to remember and told me freely "…contact, breathing" (W).<br>➢ It can be transmitted through air/ breathing, shaking hands, kissing, contact with others and when face masks are not applied properly (W).
Facility adaptation	
Training provided to HCP	➢ …. there was continuous and repeated awareness creation on the preventive measures, how they apply it to prevent COVID-19… (HCP)<br>➢ After the first case of COVID-19 was confirmed in our country, all health care providers including supportive staff were oriented about covid-19 and how to protect themselves and their clients (HCP).<br>➢ Training was given for all health professionals by trained woreda health professionals, how the health professional can use mask and keeping distance, source of the virus's transition and the like (HCP).
PPE use and social distancing	➢ All health workers have applied face masks and sanitizer while providing services (W).<br>➢ Health professionals kept all PPE materials in place while serving clients (HCP).

	➢ Health professionals have put on their face masks, enforce clients to wear face masks during facility visits and hand washing soap has also been kept in place for clients (W).<br>➢ We had arranged client sitting chairs at all departments to keep their social distance; we had assigned one personnel to educate and to keep their social distance (HCP).<br>➢ We were giving care for patients face to face in and in close contact so far, but now we are providing two meters distance (HCP).

Supplementary Table 4. *Themes (authors interpretation) and illustrative quotes of key informants on perception of client flow and barriers of community facility visit.*

Themes	Illustrative Quotes
Perception of client flow	
Client flow decreased initially and increased through time	➢ During my ANC visit, I have seen some clients receiving health services. At the beginning of coronavirus some people did not want to receive the services for fear of contracting the disease. So, client flow at that time has decreased (W).<br>➢ Following of covid-19 positive case detection in the country, somewhat patient flow was decreased HCP).<br>➢ During COVID-19 time, the patient flow has dramatically decreased at the beginning (HCP).<br>➢ Right now, the entire community members have no fear or concern about acquiring the disease (HCP).<br>➢ Becomes the same as pre-COVID-19 time since the state of emergency lifted (HCP).
Barriers for service utilization	
Fair of acquiring the disease in the facility	➢ You can have this risk at transport and at health facilities during service provision and from other clients/patients. That is the first fear (HCP).<br>➢ Health professionals subjected to additional COVID-19 related tasks, patient flow decreased due to emerging concerns and fears of contracting the disease (HCP).<br>➢ I have postponed my follow up at that time for fear of acquiring the disease from health professionals/health centers. The same is true for other clients in our area and some mothers have received their visit in private clinics as we perceived almost all staff were infected (W).<br>➢ Health workers wear face masks for themselves, but they don't let all clients wear face masks during facility visits (W).

Service deprioritized	➢ As much as possible we tried to make faster service provision for their children and give advice for them not come back frequently, they can manage themselves at home if it is easy (HCP)<br>➢ We also used tele medicine for mild cases, because at the initial phase there was a direction of avoiding hospital visits for cases other than emergency (HCP).<br>➢ Initially priority was given for patients who have cough but without compromising maternal and child health care services (HCP).
Low transportation access	➢ It is also another common reason for all of us to reduce client flow to the facility (W).<br>➢ Initially …… mothers were staying at hospital unnecessarily due to absence of transportation/ambulance/ (HCP).<br>➢ In this area there was no transport restriction, but numbers were reduced to half sit and cost was doubled. It was one of the factors to reduce flow (HCP).<br>➢ Travel restrictions are also another reason for low client flow which is more pronounced among mothers from far kebeles (HCP).
Public panic	➢ At the beginning of covid-19 occurrence, the community panicked and feared acquiring the disease (HCP).<br>➢ Our basic challenge is fear of the disease. The community heard the severity of the disease in the developed country in the media, but now the problem is solved (HCP).<br>➢ Nationally the people panicked so there was a tendency of not visiting hospitals (HCP).<br>➢ The community has been frightened of contracting the disease at the beginning (W).

BMJ Open

Impact of the COVID-19 Pandemic on Utilization of Facility-Based Essential Maternal and Child Health Services from March to August 2020 Compared to Pre-Pandemic March to August 2019: a Mixed Method Study in North Shewa Zone, Ethiopia

Journal:	*BMJ Open*
Manuscript ID	bmjopen-2021-059408.R2
Article Type:	Original research
Date Submitted by the Author:	06-May-2022

Complete List of Authors:	Bekele, Chalachew; St Paul's Hospital Millennium Medical College, HaSET MNCH Research Program<br>Bekele, Delayehu; St Paul's Hospital Millennium Medical College Department of Obstetrics and Gynecology<br>Hunegnaw, Bezawit; St Paul's Hospital Millennium Medical College, Department of Pediatrics and Child Health<br>Van Wickle, Kimiko; Harvard University T H Chan School of Public Health, Epidemiology<br>Gebremeskel, Fanos ; St Paul's Hospital Millennium Medical College, Birhan HDSS<br>Korte, Michelle ; Harvard University T H Chan School of Public Health, Department of Global Health and Population<br>Tedijanto, Christine ; University of San Francisco, Francis I. Proctor Foundation<br>Taddesse, Lisanu; Ethiopian Public Health Institute<br>Chan, Grace; Harvard Medical School, Boston Children's Hopsital, Department of Pediatrics; Harvard University T H Chan School of Public Health, Epidemiology

**Primary Subject Heading**:	Public health
Secondary Subject Heading:	Public health
Keywords:	COVID-19, Paediatric infectious disease & immunisation < PAEDIATRICS, Antenatal < GENETICS, Community child health < PAEDIATRICS, QUALITATIVE RESEARCH

SCHOLARONE™
Manuscripts

I, the Submitting Author has the right to grant and does grant on behalf of all authors of the Work (as defined in the below author licence), an exclusive licence and/or a non-exclusive licence for contributions from authors who are: i) UK Crown employees; ii) where BMJ has agreed a CC-BY licence shall apply, and/or iii) in accordance with the terms applicable for US Federal Government officers or employees acting as part of their official duties; on a worldwide, perpetual, irrevocable, royalty-free basis to BMJ Publishing Group Ltd ("BMJ") its licensees and where the relevant Journal is co-owned by BMJ to the co-owners of the Journal, to publish the Work in this journal and any other BMJ products and to exploit all rights, as set out in our licence.

The Submitting Author accepts and understands that any supply made under these terms is made by BMJ to the Submitting Author unless you are acting as an employee on behalf of your employer or a postgraduate student of an affiliated institution which is paying any applicable article publishing charge ("APC") for Open Access articles. Where the Submitting Author wishes to make the Work available on an Open Access basis (and intends to pay the relevant APC), the terms of reuse of such Open Access shall be governed by a Creative Commons licence – details of these licences and which Creative Commons licence will apply to this Work are set out in our licence referred to above.

Other than as permitted in any relevant BMJ Author's Self Archiving Policies, I confirm this Work has not been accepted for publication elsewhere, is not being considered for publication elsewhere and does not duplicate material already published. I confirm all authors consent to publication of this Work and authorise the granting of this licence.

For peer review only

Impact of the COVID-19 Pandemic on Utilization of Facility-Based Essential Maternal and Child Health Services from March to August 2020 Compared to Pre-Pandemic March to August 2019: a Mixed Method Study in North Shewa Zone, Ethiopia

Authors

Chalachew Bekele, BSc MPH[1], Delayehu Bekele, MD MPH[2], Bezawit M. Hunegnaw, MD MPH[3], Kimiko Van Wickle, BSC MSPH[4], Fanos Ashenafi, BSc MSc[1], Michelle Korte, MPH[5], Christine Tedijanto, PhD[6], Lisanu Taddesse, MD MPH[7], Grace J. Chan, MD MPH PhD[3,4,8]

Affiliations

[1]HaSET MNCH Research Program, St. Paul's Hospital Millennium Medical College, Addis Ababa, Ethiopia

[2]Department of Obstetrics and Gynecology, St. Paul's Hospital Millennium Medical College, Addis Ababa, Ethiopia

[3]Department of Pediatrics and Child Health, St. Paul's Hospital Millennium Medical College, Addis Ababa, Ethiopia

[4]Department of Epidemiology, Harvard T.H. Chan School of Public Health, Boston, MA, USA

[5]Department of Global Health and Population, Harvard T.H. Chan School of Public Health, Boston, MA, USA

[6]Francis I. Proctor Foundation, University of California San Francisco, San Francisco, CA, USA

[7]HaSET MNCH research program, Addis Ababa, Ethiopia.

[8]Division of Medical Critical Care, Boston Children's Hospital, Harvard Medical School Boston, MA, USA

Corresponding Author

1. Grace J Chan, MD MPH PhD
   677 Huntington Ave, Kresge 913, Boston, MA 02115
   grace.chan@hsph.harvard.edu
2. Chalachew Bekele, BSc MPH
   Addis Ababa, Ethiopia
   cshiferaw@hsph.harvard.edu

Word Count: 4,147 (Excluding Title, Abstract, Figure, Table, Abbreviations and References)

Abstract Word Count = 266

Abstract

Introduction: Health systems are often weakened by public health emergencies that make it harder to access health services We aimed to assess MNCH service utilization during the first six months of the COVID-19 pandemic compared to prior to the pandemic.

Methods: We conducted a mixed study design in eight health facilities that are part of the Birhan field site in Amhara, Ethiopia and compared the trend of service utilization in the first six months of COVID-19 to the corresponding time and data points of the preceding year.

Result: New family planning visits (43.2 to 28.5/month, P = 0.014) and sick under five child visits (225.0 to 139.8/month, P = .007) declined over the first six months of the pandemic compared to the same period in the preceding year. Antenatal (208.9 to 181.7/month, P = 0.433) and postnatal care (26.6 to 19.8/month, P = 0.155) visits, facility delivery rates (90.7 to 84.2/month, P = 0.776), family planning (313.3 to 273.4/month, P = 0.415) declined although this did not reach statistical significance. Routine immunization (37.0 to 36.8/month, P = 0.982) visits for children were maintained. Interviews with health care providers and clients highlighted several barriers to service utilization during COVID-19, including fear of disease transmission, economic hardship, and transport service disruptions and restrictions. Enablers of service utilization included communities' decreased fear of COVID-19, and awareness-raising activities.

Conclusion: We observed a decline in essential MNCH services particularly in sick children and new family planning visits. To improve the resiliency of fragile health systems resources are needed to continuously monitor service utilization and clients' evolving concerns during public health emergencies.

Strengths and limitations of this study

Strengths of the study:

- We presented data on service utilization during the early months of the pandemic in a rural, agrarian region in Ethiopia.
- The mixed-methods approach integrated both quantitative service utilization coverage and exploratory qualitative interviews to understand our findings and the reasons for changes in service utilization.

Limitation of the study:

- We focused on the coverage of service utilization as the primary outcome rather than mortality or morbidity rates.
- We do not have detailed data on service provision (e.g., which services were restricted and for how long, in what manner).
- Since we collected the qualitative data three months past the initial six months of the pandemic (March to August 2020), there may be recall bias.

For peer review only - http://bmjopen.bmj.com/site/about/guidelines.xhtml

INTRODUCTION

The World Health Organization (WHO) declared coronavirus disease-2019 (COVID-19) a global pandemic on 11 March 2020[1] and Ethiopia registered its first case of COVID-19 on 13 March 2020. Ethiopia has reported relatively low numbers of COVID-19 cases and COVID-related deaths, with 63,367 confirmed cases and 974 deaths in a population of 119 million, as of 10 September 2020.[2] The majority of reported cases were from the capital city, Addis Ababa, and only 365 confirmed cases and 8 deaths were registered by 30 August 2020 in the North Shewa Zone (the 3rd administration unit of the country) where the study was conducted.

Multiple preventive measures focused on social distancing and wearing masks were undertaken in Ethiopia.[3] Some health facilities were assigned as COVID-19 isolation and quarantine centers, and many health facilities suspended elective surgeries and select outpatient services. This increasing burden of managing COVID-19 on health facilities and health care providers left the health system overstretched, challenging its ability to operate effectively. As shown during the 2014-2015 Ebola outbreak in West Africa, when health systems are overwhelmed by outbreaks, mortality from vaccine-preventable and other treatable diseases can increase dramatically.[4,5]

Well-organized and equipped health systems can continue to provide equitable access to essential services through an emergency[6], but fragile health systems in developing countries face organizational and resource constraints when confronted with emergencies such as pandemics. The WHO advises nations to identify and prioritize MNCH essential services like routine vaccination, reproductive health services, childbirth, and care of young infants and older adults in their efforts to maintain continuity of service delivery and make strategic shifts to ensure limited resources provide maximum benefit for the population.[7] However, the disruption of services and diversion of resources away from essential sexual and reproductive health care due to the prioritization of the COVID-19 response are expected to increase the risks of maternal and child morbidity and mortality.[8]

A lockdown or stay-at-home policy was largely not in place in Ethiopia where the study was conducted, and health facilities were open during the study period from March to August 2020. The Ministry of Health (MOH) of Ethiopia focused on preventive measures and control of the pandemic. Possible shifts of the health workforce and the health system toward the COVID-19 response may have contributed to low utilization of routine services. For example, “half sit” policies (29 March 2020) decreased maximum occupancy on public transit by half and increased the cost of transportation when traveling within the region, while transportation between regions was paused completely. Additionally, school closures (March 2020), the declaration of a state of emergency (08 April 2020), awareness campaigns about the pandemic, and case reports, both suspected and confirmed, may have contributed to the fear of exposure to COVID-19, especially for patients visiting health facilities.

To understand these effects COVID-19 pandemic in Ethiopia, the HaSET Maternal and Child Health Research Program assessed trends in MNCH care utilization from March 2019 to August 2020 as well as health care providers’ and clients’ perceptions of the barriers to and enablers of service provision and utilization during the COVID-19 emergency. This study has paramount

For peer review only - http://bmjopen.bmj.com/site/about/guidelines.xhtml

importance in filling the evidence gap on MNCH service utilization during COVID-19, both in the Ethiopian context and other low- and middle-income countries, to prevent significant damage to the gains achieved in such areas over the past several decades.

METHODS

We conducted the study in eight health facilities in the Birhan North Shewa Zone, Amhara Region, Ethiopia. The field site was established in June 2018. The Birhan field site is a community-based continuous follow-up study of health and demographic conditions that provide up-to-date information on the catchment population and establish a population frame to nest studies. We selected all catchment health facilities for this study, including five health centers, two primary hospitals (one public and one private), and one referral hospital. These facilities provide essential MNCH services for both the rural majority population and urban population within the field site catchment and non-catchment areas.

The health centers provide ANC, PNC, delivery, abortion, routine immunization (RI), integrated management of neonatal and childhood illness (IMNCI), and FP. Each health center also has a minimum of five service extension health posts, mainly for FP and RI in each kebele (the lowest administration unit in Ethiopia), and each health post sends monthly activity reports to health centers. Two public hospitals (one primary and one referral) and one private general hospital also provide the fore mentioned essential MNCH services, except for RI, which is given mainly in health centers and catchment health posts.

Mixed phenomenological qualitative and facility-based cross-sectional study designs were employed. For the quantitative part of the study, a facility-based cross-sectional survey was conducted to assess the impact of COVID-19 on essential MNCH service provision or utilization and provider-side barriers to service provision and utilization in the Birhan filed site catchment health facilities. We interviewed 91 MNCH healthcare providers (doctors, nurses, midwives, and clinical officers available at the time of data collection) with uniformly structured questionnaires about their perception of client flow and possible barriers for respective sections. Twelve out of 91 healthcare providers were working in two MNCH departments and were interviewed twice. In addition to this, we extracted retrospective, healthcare utilization time-series data from each facility using monthly facility reports and medical registers. Retrospective facilities service statistics were collected over an 18-month period from March 2019 to August 2020 using Computer Assisted Field Editing (CAFE). We extracted data from the uniformly structured questionnaires, entered it into the Open Data Kit (ODK), and collected and uploaded the data to the ODK aggregate. The monthly facility reports, and medical registers data were collected separately. The health centers' monthly reports include services given in the health posts that are extension sites for the health center, but the facility registers are exclusively for services given in the health centers.

In addition to the cross-sectional study, we implemented a phenomenological qualitative design utilizing in-depth interviews to assess client and provider side barriers and enablers to service provision/utilization in the Birhan field site catchment health facilities. We sampled and conducted in-depth interviews until we reached theoretical saturation. For this section of the study, we

For peer review only - http://bmjopen.bmj.com/site/about/guidelines.xhtml

interviewed ten facility or department heads, and nine mothers (delivered at home or facility, had ANC, or missed ANC follow-up). An interview guide with open-ended questions was translated from English to Amharic and was used to elicit the qualitative information from informants. We conducted in-person interviews with facility or department heads and women who visited facilities during COVID-19, and women who delivered at the facilities and phone interviews with women who missed an ANC follow-up or delivered at home. With the permission of the respondents, we recorded all interviews and transcribed all records into English for further analysis. To ensure the safety of the data collectors and participants, data collectors wore masks and practiced physical distancing during training and data collection from 2–20 November 2020.

The extracted data was exported to Stata 17.0 for analysis and the average MNCH services utilization was calculated each month to quantify the changes pre-COVID-19 (March to August 2019) and during the COVID-19 (March to August 2020) pandemic. To control for potential seasonal fluctuations in service utilization, March to August 2019 and March to August 2020 were considered pre-COVID-19 and COVID-19 periods, respectively. Across all health facilities, we had 48 paired months of observations (6 months for each of 8 facilities) for all essential MNCH variables except for RI, which was only administered at the five health centers (and corresponding extension health posts), resulting in 30 paired months Errors were found in some cases where medical records were misplaced and data for some months were missing or partially filled. To avoid the effect of missing and partially filled values, analogous months' data from the same facility were excluded from the data analysis. Finally, we compared visits for each MNCH service in the pre-COVID-19 and COVID-19 period using a two-tailed independent sample t-test. We repeated the analysis for the initial two months (March to April 2020) of the pandemic and the analogous period (March to April 2019) to examine changes in service utilization at the onset of the COVID-19 pandemic. We used a significance level of $\alpha = 0.05$ for all statistical tests.

In addition to the quantitative metrics listed above, English language transcript data was entered in Dedoose software for qualitative data analysis. After familiarization with the data, the content of the data was coded line by line for thematic analysis following a framework theory approach to describe and interpret health providers' and communities' perceptions of barriers and enablers to MNCH service provision. The framework approach involves using some pre-assigned themes to initially categorize data while also adjusting and iterating the coding scheme to accommodate newly emergent themes, sub-themes, and categories through inductive interpretation.[9] Coded data were examined for potential relationships and themes were also assessed across relevant participant demographic categories to understand different user perspectives. Findings were described under pre-assigned and newly emerged themes.

Ethic Statement

This study involved human participants and the protocol was approved by the Institutional Review Board (IRB) of Saint Paul's Hospital Millennium Medical College (SPHMMC) (PM23/104) and the Harvard T.H. Chan School of Public Health (HSPH) (IRB20-1574). We obtained permission from all individual health facilities and individual verbal consent from interview respondents.

For peer review only - http://bmjopen.bmj.com/site/about/guidelines.xhtml

Patient and Public Involvement

As in-person meetings were restricted by local authorities during protocol development and data collection due to COVID-19 pandemic, we were not permitted to involve clients or the public in study design or reporting and dissemination plans of our research.

RESULTS

We extracted data from three hospitals and five health centers (includes 34 service expansion health posts in the community). Maternal health facility visits for ANC, PNC, facility delivery, and abortion-related services decreased in the time of COVID; however, we do not see a statistically significant change. The FP services utilization in the health centers and hospitals declined from 105.5 to 66.5 visits per month (P =0.051) after the onset of the pandemic. Repeat FP visits significantly declined (P=0.046) while new FP visits did not change. When combining health facilities with community health post data[10] the new FP visits declined significantly from 43.2 visits per month to 28.5 visits per month (P = 0.029) and there was no significant change in repeat FP visits.
Declines in service utilization were also observed for Integrated Management of Neonatal and Childhood Illness (IMNCI), or sick child visits, defined as a facility visit for a sick child under five years old. The mean number of IMNCI visits for sick children under 5 years old declined from 225.0 visits per month in 2019 to 139.8 visits per month in 2020 (P = 0.014). This significant decline persists for two age stratifications of IMNCI visits (2 months to under 2 years, and 2 years to under 5 years). However, there was no significant change (37.0 to 36.8/month, P = 0.982**)** in child visits for routine immunizations, including BCG, OPV-0, pentavalent (DPT-HepB-HIP) and measles vaccinations **(Table 1).** Similar results were found comparing essential MNCH service over two months during COVID and the analogous pre-COVID two-month period **(Supp Table 1)**

Ninety-one healthcare providers working in maternal, newborn, and child health services were asked about the client flow during COVID-19. Sixty-seven percent of the health care providers (HCPs) perceived that client flow decreased and 31% of them believed client flow did not change **(Supp Table 2)**. Qualitative interviews also supported the observed decrease in client flow, with descriptions of sharper contractions in service utilization in the first couple of months after the onset of the pandemic but resumed to approximately normal levels over subsequent months. In exploring the perception of lower service utilization during the initial couple of months of COVID-19 by clients of the health system and HCPs, a study in southwest Ethiopia compares the initial two months of the COVID-19 pandemic (March to April 2020) to analogous pre-COVID-19 months (March to April 2019).

Barriers to service provision and utilization during COVID-19

Even though essential MNCH service utilization was largely maintained, clients' fear of acquiring the disease from the facility, travel restrictions, increased transportation costs due to the half-seat order by the government, and fear of acquiring the disease on the way to the health facility were the main barriers for service utilization perceived by healthcare providers **(Table 2).**

Fear of contracting the disease and lack of access to transportation were the most described barriers **(Supp Table 3).** Particularly during the first few months after the onset of COVID-19 in

For peer review only - http://bmjopen.bmj.com/site/about/guidelines.xhtml

Ethiopia and the imposition of travel restrictions and other public health measures, heightened community fear of acquiring the disease and high levels of public panic were barriers to facility-based service utilization. Community members were afraid of contracting the disease in crowded spaces, on public transportation routes to facilities, and at facilities from health care workers or other patients, especially as confirmed COVID-19 cases were reported at facilities, causing further delay of care-seeking. One client said *"I have postponed my follow up at that time for fear of acquiring the disease from health professionals and health centers. The same is true for other clients in our area, and some mothers have received their visit in private clinics as we perceived almost all staff were infected"*.

The economic hardship and half-seat transportation restrictions during COVID-19 prevented some clients from being able to pay for transportation. Clients described lacking money to purchase PPE and one HCP noted that the closed market movement affected people's incomes, as reflected by patients delaying treatment until conditions were more severe or defaulting on treatment.
Lastly, multiple clients described that they might be forcibly quarantined or presumed COVID-19-positive if they were to visit facilities, and this fear also deterred facility visits. HCPs described many challenges related to the under-preparedness of the health system to manage suspected cases of COVID-19. Often these challenges manifested through physical infrastructure constraints and a shortage of guidelines for managing quarantine and isolation centers for suspected COVID-19 cases.

Enablers of service provision and utilization during COVID-19
In terms of knowledge of COVID-19, all women had heard about the disease, but a few were in doubt about the existence of COVID-19 in the area, which might be an enabling factor for facility-based service utilization. A client respondent said, "*I do not believe it exists, especially in our area. It might be real / exist in other areas/countries. Healthcare providers just suspect and take everyone into an isolation/quarantine center, even though they are healthy and free of any signs and symptoms…*". Some described that COVID-19 could not affect them because God and/or Mary will protect them, citing the importance of prayer as a protective measure.

Facility adaptations, including training for healthcare providers, hand washing facilities, physical distancing and awareness creation, and health education given by local authorities, increased client awareness of COVID-19 prevention and facility-based service utilization amid the pandemic over time **(Supp Table 4).**

Understanding Service Utilization Trends during COVID-19
The barriers and enablers highlighted in the interviews interact with each other dynamically, as depicted below. Some barriers were more substantial than others, particularly fear of the disease, transportation access, and economic-related barriers, while certain pull factors encouraged facility visits, particularly over time as fear subsided, community awareness measures were undertaken, and facilities implemented adaptations to manage both COVID-19 and routine services **(Figure 01).**

For peer review only - http://bmjopen.bmj.com/site/about/guidelines.xhtml

DISCUSSION

We examined the impact of the COVID-19 pandemic on essential MNCH service utilization by analyzing data from health facility records and healthcare providers' and patients' perspectives. In the context of already poor health outcomes, significant reductions in service utilization for maternal and child health may have substantial adverse impacts. Essential MNCH services such as family planning initiation and sick visits for under five years old significantly declined during the COVID-19 pandemic. For maternal health, FP, ANC, PNC, facility delivery, and abortion services utilization decreased, but the change was not significant during the initial six months of the pandemic likely because of a small sample size.

A modeling study of essential maternal and child health interventions across 118 low- and middle-income countries over a six-month period estimated a reduction of services by 9.8–18.5% and 39.3–51.9% in the least and most severe scenarios, respectively,[11] due to the COVID-19 pandemic and in China, health service utilization declined significantly after the outbreak and all indicators rebounded beginning in March 2020, but most had not recovered to their pre-COVID-19 levels by June 2020.[12] Other studies from African countries have reported a 37% overall healthcare utilization and 42% of visit reduction[13] and impacts on ANC, PNC, and facility delivery.[14,15,16,17] In Addis Ababa, COVID-19 confirmed cases and public panic were higher than in other areas of Ethiopia and women and children's facility-based services utilization declined.[18] Similarly, a significant reduction of essential MNCH service utilization was observed from March to June 2020 in southwest Ethiopia.[19] In contrast, FP, institutional delivery, RI, and ANC did not vary significantly between pre-COVID-19 and during COVID-19 in the Amhara region, Ethiopia[20] and in Kenya.[21] Similarly, in our study maternal facility-based healthcare provision was not significantly affected. These findings may be due to the government response to COVID-19, including absence of a stay at home/lockdown policy, relatively low numbers of confirmed cases and death, and facilities open for MNCH services throughout the study period.

Globally, contraception services were shut down or not accessible[22] and service provision declined.[23,24] In Addis Ababa, FP service utilization declined for new users and repeat users.[18] In our study health center and hospital-based family planning service utilization decreased (105.5 to 66.5/month, P = 0.051), but this may have been balanced by services provided by health extension workers at health posts in villages which when combined shows that repeat FP service utilization was maintained (313.3 to 273.4/month, P = 0.415).New FP initiation w significantly declined (43.2 to 28.5/month, P = 0.029). These results suggest that utilization of family planning services is more likely to occur in uncrowded service delivery locations that are nearby without requiring extensive travel.

For child health, the number of IMNCI visits, or sick child visits, significantly declined by 38% (225.0 to 139.8/month, P = 0.014). Similarly, child health services declined by 33% in three sub-Saharan African countries including Ethiopia due to COVID-19.[24] It is possible that the decrease in child sick visits was related to COVID-19 prevention and control activities. The leading causes of under five-year-old children morbidity in Ethiopia,[25] including acute respiratory illness, fever, and diarrhea, may have decreased due to school closures (older siblings less exposed), limited interactions with peers in the community, spending more time indoors, mask-wearing at

community gatherings, hand washing, physical distancing, and other personal protective equipment and practices. Despite a marked reduction in supply chain distribution of vaccines in Ethiopia during COVID-19, [26] we found that RI remained stable during the initial six months of the pandemic, which was different from the findings in Colombia, India, and Brazil where RI declined during the pandemic.[27-29] The MOH of Ethiopian prioritized RI, especially measles, during COVID-19. Existing health extension workers stationed at health posts for community-based services, offering hygiene and sanitation services, FP, and RI, may have sustained accessibility to these services during COVID-19 as they have close relationships with clients and health posts and are often not crowded.

At the time of data collection, early in the pandemic, respondents mainly described not feeling many tangible impacts of COVID-19 on their daily lives, so they conducted daily living activities as usual. This easing fear of COVID-19 may have enabled women to feel that they could safely attend services, but it also has important implications as the pandemic continues, particularly as cases in Ethiopia have risen substantially. Awareness and education campaigns are needed to encourage behavior change. Moreover, communities' belief that God may protect them from infection indicates the important role of engaging religious leaders as champions in behavior change campaigns. An additional key recommendation is systematically addressing misinformation and doubt to increase population compliance with preventive measures, particularly as Ethiopia faces a rising caseload, increasing prevalence of variants, and a stalled vaccine rollout that may take months or years to reach substantial population coverage. Less than 8.4% of the population has received at least one dose of the COVID-19 vaccine as of 19 February 2022.[30]

Barriers to maternal facility visits included women not wanting to bother anyone, lack of support from healthcare workers, the influence of the media,[31] lockdowns, fear of contracting the disease,[32] shift of focus towards pandemic, resource constraints[14] and non-conducive working environments for healthcare providers. [33] In addition, women experienced fears of contracting the disease, economic hardship, and lack of access to transportation. Particularly during the first few months after the onset of COVID-19 in Ethiopia, there was an imposition of travel restrictions and other public health measures like a state of emergency, and high levels of public panic. Facilities restricted some MNCH services at the beginning of the pandemic. Multiple clients described fearing that they might be forcibly quarantined or presumed COVID-19-positive if they were to visit facilities; this fear deterred facility visits. While we found that sick child visits and new family planning services were most affected by the pandemic, despite the presence of those barriers, the declines among other essential services were not as significant. Health system resilience and adaptations to maintain provision of services was demonstrated through prioritization of key interventions such as immunization and reliance on community sources of service provision such as health posts for family planning to maintain the health system.

Strengths of the study: We present primary data on service utilization during the early months of the pandemic in an area of Ethiopia, one of its agrarian regions, which is generalizable to 80% of the country's rural population.[34] We leveraged an existing research network, the HaSET MNCH Research Program (www.hasetmch.org)and our existing Birhan field site.[35] The mixed-methods approach integrated quantitative service utilization coverage data with sociocultural, contextual,

For peer review only - http://bmjopen.bmj.com/site/about/guidelines.xhtml

and exploratory qualitative interviews to understand trends in service utilization. The study highlights success stories in community-based care and government leadership for key services like routine immunization that may benefit other settings.

Limitations of the study: Our study focused on service utilization and may not have been powered to detect significant differences. We do not have detailed data on service provision. Recall bias was a potential limitation since qualitative data were collected three months after the study period (March to August 2020).

CONCLUSION

The utilization of essential MNCH services is crucial to achieving favorable health outcomes. In developing countries like Ethiopia, health systems are often too fragile to withstand the direct increase in the volume of patients and the indirect health consequences of a pandemic. Our study presents early findings on a decline in the utilization of MNCH services especially in new family planning services and sick child visits. Further study is needed to assess the effect of the pandemic on morbidity and mortality among women and children. To sustain health service utilization during challenging times such as the pandemic, resources are required by government leaders, policymakers, and clinicians to improve the resilience of their health system to monitor service utilization while at the same time engaging with providers and clients to understand and address their evolving concerns about MNCH service uptake.

ABBREVIATIONS

CAFÉ: Computer Assisted Field Editing, COVID-19: Coronavirus Disease - 2019, DPT: Diphtheria Pertussis and Tetanus, EOC: Emergency Operation center, EPHI: Ethiopian Public Health Institute, FP: Family Planning, HC: Health Center, HCP: Healthcare provider, HepB: Hepatitis B, HIP: Haemophilus influenza, HIV/AIDS: Human Immunodeficiency Virus/ Acquired Immune Deficiency Syndrome, HP: Health post, HSPH: Harvard School of Public Health, Integrated Management of Neonatal and Childhood Illness (IMNCI), IRB: Institutional Review Board, MCH: Maternal and Child Health, MNCH: Maternal, Newborn and Child Health, MOH: Ministry of Health, ODK: Open Data Kit, PNC: Postnatal care, RI: Routine immunization, RMNCH: Reproductive, Maternal, Newborn and Children Health, SPHMMC: Saint Paul Hospital Millennium Medical College,, WHO: World Health Organization.

ACKNOWLEDGEMENTS

We acknowledge the kind help and encouragement we received from the zone, woreda, and study facilities. Our deepest gratitude goes to the women and health care providers who participated in this study and to all data collectors and supervisors for their dedicated work.

CONTRIBUTORSHIP

1. Chalachew Bekele and Grace J. Chan: Equally participated in design, implementation, analysis, interpretation, write up and submission process.

2. Delayehu Bekele: Participated in design, implementation, interpretation and write up process.

3. Bezawit M. Hunegnaw: Participated in design, implementation, interpretation and write up process.

4. Kimiko Van Wickle: Participated in design, implementation, analysis, and interpretation

5. Fanos Ashenafi: Analysis, interpretation and write up process.

6. Michelle Korte: Participated in design, implementation, analysis, interpretation and write up process.

7. Christine Tedijanto: Participated in design, implementation, interpretation and write up process.

8. Lisanu Taddesse: Participated in design, implementation, interpretation and write up process.

FUNDING

This work is supported by the Bill & Melinda Gates Foundation.

GRANT/AWARD NUMBER

The research is co-funded through INV-006752 HaSET and INV-003612 ANC/PNC Innovations Platform HaSET Ethiopia Partnership.

AVAILABILITY OF DATA AND MATERIALS

The datasets used and/or analyzed during the current study are available from the corresponding author on reasonable request.

COMPETING INTERESTS

The authors declare that they have no competing interests.

References

1. **World Organization** H. Coronavirus disease (COVID-19) situation report – 162. 2020.
2. https://www.worldometers.info/coronavirus/.
3. Agency EP. Ethiopia officially declares a State of Emergency. April 9, 2020. https://www.press.et/english/p=20750.
4. Elston JW, Cartwright C, Ndumbi P, Wright J. The health impact of the 2014-15 Ebola outbreak. Public Health. Feb 2017; 143:60-70. Doi: 10.1016/j.puhe.2016.10.020.
5. Parpia AS, Ndeffo-Mbah ML, Wenzel NS, Galvani AP. Effects of Response to 2014-2015 Ebola Outbreak on Deaths from Malaria, HIV/AIDS, and Tuberculosis, West Africa. Emerg Infect Dis. Mar 2016; 22(3): 433-41. Doi:10.3201/eid2203.150977.

For peer review only - http://bmjopen.bmj.com/site/about/guidelines.xhtml

6. Organization WH. 2019 novel coronavirus (2019-nCoV): strategic preparedness and response plan. 20 February 2020.
7. Organization WH. WHO releases guidelines to help countries maintain essential health services during the COVID-19 pandemic. March 30, 2020.
8. Hall KS, Samari G, Garbers S, et al. Centring sexual and reproductive health and justice in the global COVID-19 response. Lancet. Apr 11, 2020; 395(10231):1175-1177. Doi:10.1016/s0140-6736(20)30801-1.
9. Lacey A, Donna Luff. Trent Focus: Qualitative Data Analysis. 2001.
10. Endriyas M, Alano, A., Mekonnen, E. et al. Understanding performance data: health management information system data accuracy in Southern Nations Nationalities and People's Region, Ethiopia.: BMC Health Serv Res 19, 2019.
11. Roberton T, Carter ED, Chou VB, et al. Early estimates of the indirect effects of the COVID-19 pandemic on maternal and child mortality in low-income and middle-income countries: a modelling study. Lancet Glob Health. Jul 2020;8(7): e901-e908. Doi:10.1016/s2214-109x(20)30229-1.
12. Hong Xiaoa b, XDc, d, Bradley H. Wagenaar e,f, FL, OA, h,, Yan Guoi, JMUa. The impact of the COVID-19 pandemic on health services utilization in China: The Lancet Regional Health - Western Pacific. March 24, 2021;9,100122, Apr 01, 2021. Doi: https://doi.org/10.1016/j.lanwpc.2021.100122.
13. Moynihan R SS, Michaleff ZA, et al. Impact of COVID-19 pandemic on utilization of healthcare services: a systematic review. BMJ Open; 2021 Mar 16.
14. Ahmed T, Rahman AE, Amole TG, et al. The effect of COVID-19 on maternal newborn and child health (MNCH) services in Bangladesh, Nigeria and South Africa: call for a contextualised pandemic response in LMICs. Int J Equity Health. Mar 15, 2021; 20(1):77. Doi:10.1186/s12939-021-01414-5
15. Wanyana D, Wong R, Hakizimana D. Rapid assessment on the utilization of maternal and child health services during COVID-19 in Rwanda. Public Health Action. Mar 21, 2021;11(1):12-21. Doi:10.5588/pha.20.0057
16. Ameyaw EK, Ahinkorah, B.O., Seidu, AA. et al. Impact of COVID-19 on maternal healthcare in Africa and the way forward. 201;223 (2021). Doi: https://doi.org/10.1186/s13690-021-00746-6
17. Burt JF OJ, Lubyayi L ea. Indirect effects of COVID-19 on maternal, neonatal, child, sexual and reproductive health services in Kampala, Uganda. BMJ Global Health. 2021;6: e006102. Doi:10.1136/ bmjgh-2021-006102

For peer review only - http://bmjopen.bmj.com/site/about/guidelines.xhtml

18. Gebreegziabher SB, Marrye SS, Kumssa TH, Merga KH, Feleke AK, Dare DJ, Hallström IK, Yimer SA, Shargie MB. Assessment of maternal and child health care services performance in the context of COVID-19 pandemic in Addis Ababa, Ethiopia: evidence from routine service data. Reprod Health. 2022 Feb 14;19(1):42. doi: 10.1186/s12978-022-01353-6. PMID: 35164776; PMCID: PMC8842853.
19. Kassie A, Wale A, Yismaw W. Impact of Coronavirus Diseases-2019 (COVID-19) on Utilization and Outcome of Reproductive, Maternal, and Newborn Health Services at Governmental Health Facilities in Southwest Ethiopia, 2020: Comparative Cross-Sectional Study. Int J Womens Health. 2021; 13:479-488. Doi:10.2147/ijwh.s309096
20. Wendemagegn Enbiale SGA, Meaza Seyum, Dereje Bedanie Hundie, Kassawmar Angaw Bogale, Koku Sisay Tamirat, Mulat Birhanu Feleke, Muluken Azage, Dabere Nigatu, and Henry J. C. de Vries. Effect of the COVID-19 Pandemic Preparation and Response on Essential Health Services in Primary and Tertiary Healthcare Settings of Amhara Region, Ethiopia. Am J Trop Med Hyg. 2021;105(5):1240–1246. Doi:10.4269/ajtmh.21-0354
21. Shikuku1 D, IN, SG, et al. Early indirect impact of COVID-19 pandemic on utilization and outcomes of reproductive, maternal, newborn, child, and adolescent health services in Kenya. medRxiv; 2021.
22. Contraception and COVID-19. Disrupted supply and access. 2020 April 15.
23. Paulo Henrique das Neves Martins Pires CM, Ahmed Abdirazak, Jaibo Rassul Mucufo, Martins Abudo Mupueleque DZ, Ronald Siemens and Celso Fernando Belo. Covid-19 pandemic impact on maternal and child health services access in Nampula, Mozambique: mixed methods research. BMC Health Serv Res 21, 860 (2021). Doi: https://doi.org/10.1186/s12913-021-06878-3
24. Nega Assefa AS, Dongqing Wang, Michelle L. Korte, Elena C. Hemler, Yasir Y. Abdullahi, Bruno Lankoande, Ourohire Millogo, Angela Chukwu, Firehiwot Workneh, Phyllis Kanki, Till Baernighausen, Yemane Berhane, Wafaie W. Fawzi,and Ayoade Oduola. Reported Barriers to Healthcare Access and Service Disruptions Caused by COVID-19 in Burkina Faso, Ethiopia, and Nigeria: A Telephone Survey. Am J Trop Med Hyg. 2021;105(2):323–330. Doi:10.4269/ajtmh.20-1619
25. ICF CSACEa. Ethiopia Demographic and Health Survey 2016. Addis Ababa, Ethiopia, and Rockville, Maryland, USA: CSA and ICF2016.
26. Nigus M ZM, Abraham K, Shiferaw A, Admassu M, Masresha B. Implementing nationwide measles supplemental immunization activities in Ethiopia in the context of COVID-19: process and lessons learnt.: Pan Afr Med J. 2020. p. 36.

27. Moreno-Montoya J BS, Rojas Sotelo JC. Impact of the COVID-19 pandemic on routine childhood immunization in Colombia, Archives of Disease in Childhood. 20 July 2021.
28. Radhika Jain a ACb, Camille Falézan a, Mustufa Patel b, Pascaline Dupas a. COVID-19 related immunization disruptions in Rajasthan, India: A retrospective observational study,. p. 4343–4350.
29. Matos CCdSA, Barbieri, C. L. A., & Couto, M. T. Covid-19 and its impact on immunization programs: reflections from Brazil.: Revista De Saúde Pública , 54 , 114; 2020.
30. World health Organization (WHO) Coronavirus (COVID-19) Dashboard with vaccination data.
31. Karavadra B, Stockl, A., Prosser-Snelling, E. et al. Women's perceptions of COVID-19 and their healthcare experiences: a qualitative thematic analysis of a national survey of pregnant women in the United Kingdom. BMC Pregnancy Childbirth; 2020.
32. Goyal M SP, Singh K, Shekhar S, Agrawal N, Misra S. The effect of the COVID-19 pandemic on maternal health due to delay in seeking health care: Experience from a tertiary center.: Int J Gynaecol Obstet; 2021 Feb.
33. Kayiga H GD, Amuge PM, Ssemata AS, Nanzira RS, Nakimuli A. Lived experiences of frontline healthcare providers offering maternal and newborn services amidst the novel corona virus disease 19 pandemic in Uganda: A qualitative study.: PLoS One; 10 Dec 2021.
34. Ethiopia Demographics 2020 (Population, Age, Sex, Trends) - Worldometer (worldometers.info).
35. Bekele D, Hunegnaw BM, Bekele C, et al. Cohort profile: the Birhan Health and Demographic Surveillance System. medRxiv. 2021:2021.01.21.21249989. doi:10.1101/2021.01.21.21249989

Tables

Table 1. *Comparing essential MNCH service utilization over six months between COVID-19 (Mar-Aug 2020) and analogous pre-COVID-19 (Mar-Aug 2019) periods.*

Visit Type	Mean number of visits/month over six months	t-statistic	p-value	Lower p-value	Upper p-value	Paired obser

	2019	2020			†	††	vation s
**I. Maternal visit**	**376.3**	**321.2**	**1.11**	**0.270**	**0.865**	**0.135**	**48/48**
1. Antenatal care	208.9	181.7	0.79	0.433	0.784	0.216	40/48
2. Postnatal care	26.6	19.8	1.44	0.155	0.922	0.078*	30/48
3. Facility delivery	90.7	84.2	0.29	0.776	0.612	0.388	41/48
4. Abortion-related services	11.8	9.8	0.56	0.578	0.711	0.289	34/48
5. Overall FP services in health posts, health centers and hospitals	313.3	273.4	0.82	0.415	0.792	0.207	47/48
5.1 New FP services	43.2	28.5	1.22	0.029	0.986	0.014**	47/48
5.2 Repeat FP services	270.2	244.9	0.57	0.567	0.716	0.284	47/48
6. FP services in health centers and hospitals	105.5	66.5	1.99	0.051	0.974	0.026**	33/48
6.1 New FP services	8.9	7.1	0.84	0.406	0.797	0.203	33/48
6.2 Repeat FP services	96.5	59.3	1.03	0.046	0.977	0.023**	33/48
6.3 Unclassified FP services	17.7	1.3	1.12	0.039	0.981	0.019**	26/48
**II. Sick child visit (0-5years))**	**225.0**	**139.8**	**1.51**	**0.014**	**0.993**	**0.007*** **	46/48
1. MNCI Visit (< 2 months)	10.8	7.7	0.82	0.412	0.794	0.206	46/48
2. IMNCI Visit (2 months – 2 year)	101.6	50.4	1.68	0.009	0.996	0.004***	46/48
3. IMNCI Visit (2 year – 5 year)	111.6	81.8	1.15	0.034	0.983	0.017**	46/48
**III. Routine Immunization visit**	**37.0**	**36.8**	**0.02**	**0.982**	**0.509**	**0.491**	23/30
1. BCG Vaccine	31.4	36.5	-0.39	0.701	0.350	0.650	30/30
2. Oral Polio (0) Vaccine	3.2	1.0	0.88	0.384	0.808	0.192	23/30

3. Pentavalent (DPT-HepB-HIP) (all types)	100.4	101.5	-0.05	0.958	0.479	0.521	30/30
4. Measles – 1	10.3	27.5	-1.99	0.051	0.026	0.974	30/30
5. Vitamin A Dose	8.6	5.7	0.67	0.506	0.747	0.253	14/30
**Other types of visits**							
All visits	2568.9	2606.7	-0.05	0.956	0.478	0.522	48/48
Adult Outpatient Visit	2121.2	2239.7	-0.17	0.868	0.434	0.566	44/48

* $p < 0.10$ ** $p < 0.05$ *** $p < 0.01$, † Lower-tailed test: mean number of visits H_1: $\mu_{2019} < \mu_{2020}$, †† Upper-tailed test: mean number of visits H_1: $\mu_{2019} > \mu_{2020}$

Table 2. *Possible barriers to service utilization in the time of COVID-19 based on healthcare providers' perception.*

**Possible barriers to service utilization in the time of COVID-19**	**Count**	**%**
1. Fear of acquiring the diseases from the facility	97	94
2. Travel restrictions	90	87
3. Increased transportation cost (due to half sit order by the government)	89	86
4. Fear of acquiring the disease on the way to the health facility	86	83
5. Lack of transport to the HP/HC site	72	70
6. Lack of PPE for clients	67	65
7. Clients' perception of limited implementation of protective measures by healthcare providers	58	56
8. Healthcare providers advice to stay at home	54	52
9. Limited-service hours or absence of health care workers	17	17
10. Unavailability of ambulance	7	7
11. Unavailability of healthcare providers in facilities to provide outreach services.	7	7
Total interviews	**103**	

Note: *Twelve healthcare providers were working in different departments and asked twice.*

For peer review only - http://bmjopen.bmj.com/site/about/guidelines.xhtml

Figure 1: Enabling (pull) factors and barriers (push factors) for service utilization highlighted in the qualitative interviews.

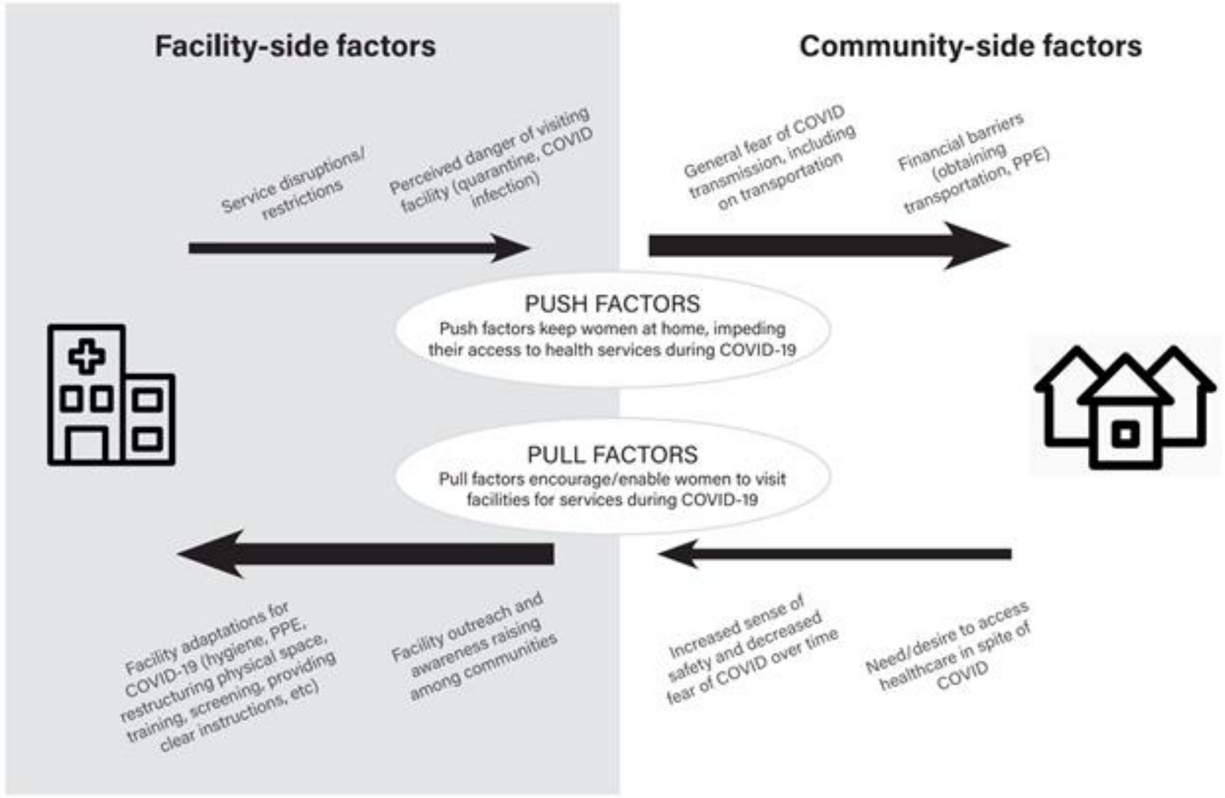

For peer review only

Supplementary Tables

Supplementary Table 1. Comparing essential MNCH service utilization over two months between COVID (April- May 2020) and analogous pre-COVID (April-May 2019) periods.

Visit Type	Mean number of visits/ months over six months		t-statistic	p-value	Lower p-value	Upper p-value	Number of paired observations
	2019	2020					
I. Maternal visit	331.3	327.3	0.05	0.959	0.520	0.480	16
1. Antenatal care	198.0	176.5	0.39	0.702	0.649	0.351	13
2. Postnatal care	17.8	20.7	-0.30	0.768	0.384	0.616	9
3. Facility delivery	85.6	87.0	-0.04	0.970	0.485	0.515	14
4. FP related services	84.1	95.1	-0.38	0.712	0.356	0.644	10
5. FP related services (hospitals, HCs and HPs combined)	289.4	227.8	0.86	0.398	0.801	0.199	16
6. Abortion-related services	10.5	10.7	-0.05	0.964	0.482	0.518	11
II. Sick child visit *(0-5years)*	201.0	126.6	1.68	0.103	0.948	0.052*	15
1. IMCI Visit (< 2 months)	7.3	3.3	1.21	0.235	0.882	0.118	15
2. IMNCI Visit (2 months – 2 year)	105.0	77.4	1.54	0.134	0.933	0.067*	15
3. IMNCI Visit (2 year – 5 year)	89.7	45.9	1.43	0.164	0.918	0.082*	15
III. Routine Immunization visit	41.0	38.1	0.16	0.875	0.563	0.437	7
1. BCG Vaccine	28.0	36.9	-0.40	0.695	0.347	0.653	10

2. Oral Polio (0) Vaccine	3.4	1.0	0.61	0.556	0.722	0.278	7
3. Pentavalent (DPT-HepB-HIP) (all types)	100.4	101.4	-0.03	0.978	0.489	0.511	10
4. Measles – 1	5.6	28.3	-1.45	0.163	0.082*	0.918	10
5. Vitamin A Dose (any dose)	6.0	1.0	0.87	0.419	0.791	0.209	4
Other types of visits							
4. All visits	2031.9	2323.5	-0.27	0.787	0.394	0.606	16
5. Adult Outpatient Visit	1811.6	2147.5	-0.29	0.773	0.386	0.614	14
* p < 0.10 ** p < 0.05 *** p < 0.01, † Lower-tailed test: mean number of visits H_1: $\mu_{2019} < \mu_{2020}$, †† Upper-tailed test: mean number of visits H_1: $\mu_{2019} > \mu_{2020}$							

Supplementary Table 2: Healthcare providers' perception about MNCH clients flow to the facility in the time of COVID-19.

Response	Count	Percent
Increasing	2	2%
The same	28	31%
Decreasing	61	67%
Total	91	100%

Supplementary Table 3. *Themes (authors interpretation) and illustrative quotes of key informants on factors enabling community facility visits during COVID-19.*

Themes	Illustrative Quotes
COVID-19 perception	
People are in doubt about COVID-19 existence in the area.	➢ I do not believe it exists, especially in our area. It might be real / exist in other areas/countries. They just suspect and take everyone into an isolation/quarantine center, but they are healthy and free of any signs and symptoms…. (Women)

	➢ ….. I have never seen anyone with such a real problem in our area. We have heard about it on radio and TV, so I found it difficult to believe and I do not believe it is real (Women).<br>➢ There are huge gaps, misconceptions, and challenges in practical preventive practices. They even perceived that the disease may not be real. Clients recovered from COVID-19 without any sign and symptom disseminated the information to the community and based on that the community misconceived that the virus might not be real from the beginning (HCP).<br>➢ Right now, the entire community members have no fear or concern about acquiring the disease ……. we are not concerned about client decrement related to COVID-19. Specially after the 5 months state of emergency was lifted things are returned to pre-COVID time, …. (HCP).
No/Low COVID-19 impact perception on daily life	➢ COVID was for outsiders not for us, it was for political issues, the machine for COVID test was false (Women).<br>➢ Has COVID-19 been affecting your life in any way? P: No nothing (Women)<br>➢ I do not think we are at risk because we are not getting out of home most of the time and living in rural areas without any contact (Women).
Knowledge on transmissio n methods	➢ Crowding at one place like the market and public transportation (Women).<br>➢ She laughed. "Media expresses it well; we know well it is also an infected person who can transmit it …" it was not on her tip of tongue she encouraged simply to remember and told me freely "…contact, breathing" (Women).<br>➢ It can be transmitted through air/ breathing, shaking hands, kissing, contact with others and when face masks are not applied properly (Women).
Facility adaptation	
Training provided to HCP	➢ …. there was continuous and repeated awareness creation on the preventive measures, how they apply it to prevent COVID-19… (HCP)<br>➢ After the first case of COVID-19 was confirmed in our country, all health care providers including supportive staff were oriented about covid-19 and how to protect themselves and their clients (HCP).<br>➢ Training was given for all health professionals by trained woreda health professionals, how the health professional can use mask and keeping distance, source of the virus's transition and the like (HCP).
PPE use and social distancing	➢ All health workers have applied face masks and sanitizer while providing services (Women).<br>➢ Health professionals kept all PPE materials in place while serving clients (HCP).

For peer review only - http://bmjopen.bmj.com/site/about/guidelines.xhtml

	➢ Health professionals have put on their face masks, enforce clients to wear face masks during facility visits and hand washing soap has also been kept in place for clients (Women).<br>➢ We had arranged client sitting chairs at all departments to keep their social distance; we had assigned one personnel to educate and to keep their social distance (HCP).<br>➢ We were giving care for patients face to face in and in close contact so far, but now we are providing two meters distance (HCP).

Supplementary Table 4. *Themes (authors interpretation) and illustrative quotes of key informants on perception of client flow and barriers of community facility visit.*

Themes	Illustrative Quotes
Perception of client flow	
Client flow decreased initially and increased through time	➢ During my ANC visit, I have seen some clients receiving health services. At the beginning of coronavirus some people did not want to receive the services for fear of contracting the disease. So, client flow at that time has decreased (Women).<br>➢ Following of covid-19 positive case detection in the country, somewhat patient flow was decreased HCP).<br>➢ During COVID-19 time, the patient flow has dramatically decreased at the beginning (HCP).<br>➢ Right now, the entire community members have no fear or concern about acquiring the disease (HCP).<br>➢ Becomes the same as pre-COVID-19 time since the state of emergency lifted (HCP).
Barriers for service utilization	
Fair of acquiring the disease in the facility	➢ You can have this risk at transport and at health facilities during service provision and from other clients/patients. That is the first fear (HCP).<br>➢ Health professionals subjected to additional COVID-19 related tasks, patient flow decreased due to emerging concerns and fears of contracting the disease (HCP).<br>➢ I have postponed my follow up at that time for fear of acquiring the disease from health professionals/health centers. The same is true for other clients in our area and some mothers have received their visit in private clinics as we perceived almost all staff were infected (Women).<br>➢ Health workers wear face masks for themselves, but they don't let all clients wear face masks during facility visits (Women).

Service deprioritized	➢ As much as possible we tried to make faster service provision for their children and give advice for them not come back frequently, they can manage themselves at home if it is easy (HCP)<br>➢ We also used tele medicine for mild cases, because at the initial phase there was a direction of avoiding hospital visits for cases other than emergency (HCP).<br>➢ Initially priority was given for patients who have cough but without compromising maternal and child health care services (HCP).
Low transportation access	➢ It is also another common reason for all of us to reduce client flow to the facility (Women).<br>➢ Initially …… mothers were staying at hospital unnecessarily due to absence of transportation/ambulance/ (HCP).<br>➢ In this area there was no transport restriction, but numbers were reduced to half sit and cost was doubled. It was one of the factors to reduce flow (HCP).<br>➢ Travel restrictions are also another reason for low client flow which is more pronounced among mothers from far kebeles (HCP).
Public panic	➢ At the beginning of covid-19 occurrence, the community panicked and feared acquiring the disease (HCP).<br>➢ Our basic challenge is fear of the disease. The community heard the severity of the disease in the developed country in the media, but now the problem is solved (HCP).<br>➢ Nationally the people panicked so there was a tendency of not visiting hospitals (HCP).<br>➢ The community has been frightened of contracting the disease at the beginning (Women).

For peer review only - http://bmjopen.bmj.com/site/about/guidelines.xhtml
